# G protein-coupled receptors: structure- and function-based drug discovery

**DOI:** 10.1038/s41392-020-00435-w

**Published:** 2021-01-08

**Authors:** Dehua Yang, Qingtong Zhou, Viktorija Labroska, Shanshan Qin, Sanaz Darbalaei, Yiran Wu, Elita Yuliantie, Linshan Xie, Houchao Tao, Jianjun Cheng, Qing Liu, Suwen Zhao, Wenqing Shui, Yi Jiang, Ming-Wei Wang

**Affiliations:** 1 The National Center for Drug Screening, Shanghai Institute of Materia Medica, Chinese Academy of Sciences, 201203 Shanghai, China; 2 The CAS Key Laboratory of Receptor Research, Shanghai Institute of Materia Medica, Chinese Academy of Sciences, 201203 Shanghai, China; 3 School of Basic Medical Sciences, Fudan University, 200032 Shanghai, China; 4 University of Chinese Academy of Sciences, 100049 Beijing, China; 5 iHuman Institute, ShanghaiTech University, 201210 Shanghai, China; 6 School of Life Science and Technology, ShanghaiTech University, 201210 Shanghai, China; 7 School of Pharmacy, Fudan University, 201203 Shanghai, China

**Keywords:** Drug discovery, Target validation

## Abstract

As one of the most successful therapeutic target families, G protein-coupled receptors (GPCRs) have experienced a transformation from random ligand screening to knowledge-driven drug design. We are eye-witnessing tremendous progresses made recently in the understanding of their structure–function relationships that facilitated drug development at an unprecedented pace. This article intends to provide a comprehensive overview of this important field to a broader readership that shares some common interests in drug discovery.

## Introduction

**Fig. 1:**
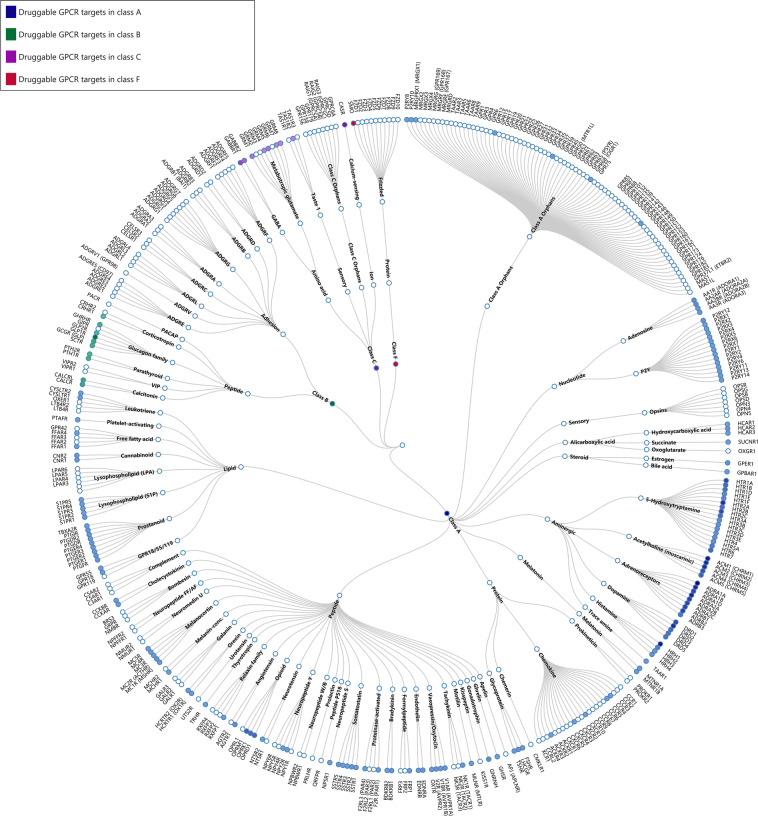
Phylogenetic tree of GPCRs as drug targets. Node represents GPCR named according to its gene name. Receptors with approved drugs on the market are highlighted by color. GPCRs are organized according to GPCR database.^4^ Approved drug list was derived from previous publications,^4,11^ complemented by additional search of newly approved entities at Drugs@FDA (accessdata.fda.gov) until June 2020. See Table S2 for details

G protein-coupled receptors (GPCRs) represent the largest protein family encoded by the human genome. Located on the cell membrane, they transduce extracellular signals into key physiological effects.^1^ Their endogenous ligands include odors, hormones, neurotransmitters, chemokines, etc., varying from photons, amines, carbohydrates, lipids, peptides to proteins. GPCRs have been implicated in a large number of diseases, such as type 2 diabetes mellitus (T2DM), obesity, depression, cancer, Alzheimer’s disease, and many others.^2^ Activated by external signals through coupling to different G proteins or arrestins, GPCRs elicit cyclic adenosine 3,5-monophosphate (cAMP) response, calcium mobilization, or phosphorylation of extracellular regulated protein kinases 1/2 (pERK1/2).^3^ The seven-transmembrane protein property endows them easy to access, while the diversified downstream signaling pathways make them attractive for drug development.^4^ The human GPCR family is divided into classes A (rhodopsin), B (secretin and adhesion), C (glutamate), and F (Frizzled) subfamilies according to their amino acid sequences (Fig. 1). Of the 826 human GPCRs, approximately 350 non-olfactory members are regarded as druggable and 165 of them are validated drug targets (Fig. 1 and Table S1).^4–6^ Latest statistical data indicate that 527 Food and Drug Administration (FDA)-approved drugs^4^ and ∼60 drug candidates currently in clinical trials target GPCRs (Table S1).^5^

Started with crystal structure determination and accelerated by cryo-electron microscopy (cryo-EM) technology, three-dimensional (3D) structural studies on a variety of GPCRs in complex with ligands, G proteins/arrestins, or both^7–10^ (involving 455 structures from 82 different receptors) significantly deepened our knowledge of molecular mechanisms of signal transduction. Novel insights into ligand recognition and receptor activation are gained from inactive, transitional, active, and *apo* states, thereby offering new opportunities for structure-based drug design (SBDD).^11^ Pharmacological parameters such as cAMP accumulation, calcium flux, ERK phosphorylation, arrestin recruitment, and G protein interaction,^12,13^ are commonly used to evaluate ligand action and biased signaling. Ligand-binding kinetics and signaling timing render another dimension for interpreting signal bias profiles and link in vitro bioactivities with in vivo effects.^14^ In this process, a series of biased and allosteric modulators were discovered by rational design, ligand screening, and pharmacological assessment leading to the identification of novel binding sites or action modes.^15,16^

Apart from crystallography and cryo-EM, the striking advancement in GPCR biology is also attributable to the deployment of powerful technologies such as nuclear magnetic resonance (NMR), hydrogen–deuterium exchange (HDX), fluorescence resonance energy transfer, bioluminescence resonance energy transfer, surface plasmon resonance, single molecule fluorescence, CRISPR/Cas9, artificial intelligence, etc. This review systematically summarizes the latest information on this important drug target family to cover both basic and translational sciences in the context of drug discovery and development.

## GPCR as drug target

### Class A

Class A GPCRs, the so called “rhodopsin-like family” consisting of 719 members, are divided into several subgroups: aminergic, peptide, protein, lipid, melatonin, nucleotide, steroid, alicarboxylic acid, sensory, and orphan.^17^ They have a conventional transmembrane domain (TMD) that forms ligand-binding pocket and additional eight helices with a palmitoylated cysteine at the C terminal.^18,19^ Given the wide range of their physiological functions, this class of receptors is the most targeted therapeutically among all other classes. By manually curating Drugs@FDA original New Drug Application (NDA) and Biologic License Application (BLA) database (data extracted from August 2017 to June 2020) and cross-referencing with Drugbank,^20^ IUPHAR and ChemBL databases, we were able to find the approved drugs associated with this class.

Over 500 GPCR drugs target class A and many of them act at >1 receptor: 75% are made against aminergic receptors and 10% for peptidic ligand receptors with indications ranging from analgesics, allergies, cardiovascular diseases, hypertension, pulmonary diseases, depression, migraine, glaucoma, Parkinson’s disease to schizophrenia, cancer-related fatigue, etc. Approximately 500 novel drug candidates are in clinical trials. Of them, 134 are for peptide-activated GPCRs, while small molecules still occupy the majority. It is noted that 6% of class A members are sensory and alicarboxylic acid receptors that have broad untapped therapeutic potentials (Table S1). Chemokine, prostanoid and melanocortin receptors constitute >8% clinical trial targets in this class.

In the past 3 years, about 20 NDAs were approved targeting mostly peptide and aminergic receptors (Table 1). Siponimod and ozanimod provide alternatives to fingolimod (approved in 2010) for treating relapsing forms of multiple sclerosis by modulating sphingosine-1-phosphate receptor. Two radiolabeled ligands, gallium 68 dotatoc and lutetium 177 dotatate, have been approved for neuroendocrine tumor and pancreatic gastrointestinal cancer diagnosis, respectively. Pitolisant, a selective inverse agonist of histamine receptor, is used to treat narcolepsy-related daytime sleepiness, while lemborexant, an orexin receptor antagonist, is used for insomnia management. Gilteritinib (ASP2215) is a small molecule inhibitor of tyrosine kinase. However, it also antagonizes serotonin receptors without any reported pharmacological consequences. Revefenacin is a long-acting antagonist of muscarinic acetylcholine receptors (mAChRs) indicated for chronic obstructive pulmonary disease. Amisulpride, trialed for antiemetic and schizophrenia, was finally approved for antiemetic in 2020. This molecule is acting as an antagonist against dopamine and serotonin receptors. Fosnetupitant, a prodrug of netupitant, was approved for chemotherapy-induced nausea and vomiting. Cysteamine treats radiation sickness via modifying action of neuropeptide Y receptor. Cannabidiol is one the active constituents of the *Cannabis* plant and was trialed for schizophrenia, graft versus host disease, and anticonvulsant. It was eventually approved in 2018 for the treatment of severe forms of epilepsy—Lennox–Gastaut syndrome and Dravet syndrome. Meanwhile, fostamatinib, indicated for chronic immune thrombocytopenia, targets >300 receptors and enzymes, including adenosine receptor A3.

**Table 1:** Newly approved drugs targeting class A GPCRs in the past 3 years

Drug	Brand name (manufacturer)	Indication	Target	GPCR class	FDA approval date
Gilteritinib	Xospata (Astellas)	Relapsed or refractory acute myeloid leukemia	Serotonin receptors	A, aminergic, 5-hydroxytryptamine	11/28/2018
Lasmiditan	Reyvow (Eli Lilly)	Migraine	HTR1F	A, aminergic, 5-hydroxytryptamine	10/11/2019
Revefenacin	Yupelri (Mylan Ireland)	Chronic obstructive pulmonary disease	CHRM1-CHRM5	A, aminergic, acethylcholine	11/09/2018
Lumateperone	Caplyta (Intra-Cellular)	Schizophrenia	HTR2A, DRD1, DRD2	A, aminergic, dopamine, 5-hydroxytryptamine	12/20/2019
Amisulpride	Barhemsys (Acacia Pharma)	Surgery-induced nausea and vomiting prevention	DRD2, DRD3, HTR7, HTR2A	A, aminergic, dopamine, 5-hydroxytryptamine	02/26/2020
Pitolisant	Wakix (Harmony)	Narcolepsy excessive daytime sleepiness	HRH3	A, aminergic, histamine	08/14/2019
Angiotensin II	Giapreza (La Jolla Pharma)	Septic vasoconstrictor for adults	AGTR1	A, peptide, angiotensin	12/21/2017
Macimorelin	Macrilen (Novo Nordisk)	Diagnosis of adult growth hormone deficiency	GHSR	A, peptide, ghrelin	12/20/2017
Elagolix	Orilissa (Abbvie)	Endometriosis-associated moderate-to-severe pain	GNRHR	A, peptide, gonadotropin	07/23/2018
Cysteamine	Procysbi (Horizon Pharma)	Radiation sickness	NPY2R	A, peptide, neuropeptide Y	02/14/2020
Lemborexant	Dayvigo (Eisai)	Insomnia	HCRTR1	A, peptide, orexin	12/20/2019
Gallium 68 dotatoc	NA (UIHC-PET Imaging Center)	Diagnostic agent for neuroendocrine tumors	SSTR2	A, peptide, somatostatin	08/21/2019
Lutetium 177 dotatate	Lutathera (AAA USA)	Gastroenteropancreatic neuroendocrine tumors	SSTR2	A, peptide, somatostatin	01/26/2018
Fosnetupitant/palonosetron	Akynzeo (Helsinn Hlthcare)	Chemotherapy-associated nausea and vomiting prevention	TACR1	A, peptide, tachykinin	04/19/2018
Mogamulizumab-kpkc	Poteligeo (Kyowa Kirin)	Non-Hodgkin lymphoma	CXCR4	A, protein, chemokine	08/08/2018
Siponimod	Mayzent (Novartis)	Relapsing forms of multiple sclerosis	S1PR1, S1PR5	A, lipid, lysophospholipid	03/26/2019
Ozanimod	Zeposia (Celgene)	Relapsing forms of multiple sclerosis	S1PR1, S1PR5	A, lipid, lysophospholipid	03/25/2020
Cannabidiol	Epidioloex (GW Research)	Epilepsy	CNR1	A, lipid, cannabinoid	06/25/2018
Latanoprostene bunod	Vyzulta (Bausch and Lomb)	Glaucoma or ocular hypertension	PTGFR	A, lipid, prostanoid	11/02/2017
Istradefylline	Nourianz (Kyowa Kirin)	Parkinson’s disease	ADORA2A	A, nucleotide, adenosine	08/27/2019
Fostamatinib	Tavalisse (Rigel Pharma)	Chronic immune thrombocytopenia	Multiple targets, including ADORA3	A, nucleotide, adenosine	04/17/2018

The drugs listed above were identified manually from Drugs@FDA original NDA and BLA database (data extracted from August 2017 to June 2020) and cross-referenced with Drugbank,^20^ IUPHAR, and ChemBL databases

*ADORA2A (A*_*2A*_*R)* adenosine A2a receptor, *ADORA3 (A*_*3*_*AR)* adenosine A3 receptor, *AGTR1 (AT1R)* angiotensin II receptor type 1, *CHRM1 (M1R)* muscarinic acetylcholine receptor M1, *CHRM5 (M5R)* muscarinic acetylcholine receptor M5, *CNR1 (CB1)* cannabinoid receptor 1, *CXCR4* C-X-C chemokine receptor type 4, *DRD1–DRD3* D1–D3 dopamine receptor, *GHSR* growth hormone secretagogue receptor, *GNRHR* gonadotropin-releasing hormone receptor, *HCRTR1 (OX*_*1*_*R)* orexin receptor type 1, *HRH3* histamine H3 receptor, *HTR1F* 5-hydroxytryptamine receptor 1F, *HTR2A (5-HT*_*2A*_*)* 5-hydroxytryptamine receptor 2A, *HTR7* 5-hydroxytryptamine receptor 7, *NPY2R* neuropeptide Y receptor Y2, *PTGFR* prostaglandin F receptor, *S1PR1* sphingosine 1-phosphate receptor 1, *S1PR5* sphingosine 1-phosphate receptor 5, *SSTR2* somatostatin receptor 2, *TACR1 (NK1R)* substance-P receptor

### Class B

This class of GPCRs is divided into two subfamilies: secretin (B1) and adhesion (B2), containing 15 and 33 members, respectively.^4,21^ Secretin subfamily members are characteristic of large extracellular domains (ECDs) and bind to vasoactive intestinal peptide (VIP), pituitary adenylate cyclase-activating peptide (PACAP), corticotropin-releasing factor (CRF), parathyroid peptide hormone (PTH), growth hormone-releasing hormone (GHRH), calcitonin gene-related peptide (CGRP), glucagon, and glucagon-like peptides (GLPs), respectively. Adhesion subfamily has nine subgroups, possessing unique N-terminal motifs, such as epidermal growth factor, cadherin, and immunoglobulin domains. They are distinguished from other GPCRs due to their roles in cell adhesion and migration.^22,23^ Apart from the long N-terminal domain, other unique features of the B2 subfamily are the GPCR autoproteolysis-inducing domain and the proteolysis site that are responsible for signaling activation through a Stachel sequence (a tethered agonist) and producing N-terminal fragment (NTF) and C-terminal fragment. The hallmarks of the B2 GPCR subfamily are a two-step activation model, the ligand–NTF interaction and the Stachel signaling/basal activity. Adhesion receptors can also signal independently of fragment dissociation and this has complicated pharmacological consequences.^22,24,25^

In this class, receptors of glucagon family peptides, followed by CGRP, PTH, GHRH, CRF, VIP, and PACAP, constitute major targets for therapeutic intervention (Table S1) of various diseases, including obesity, T2DM, osteoporosis, migraine, depression, and anxiety.^26,27^

To date, multiple GLP-1 receptor (GLP-1R) agonists have been developed by a combination of selective amino acid substitutions, enzymatic cleavage blockade, and conjugation to entities that increase binding to plasma proteins. These methods not only slow down fast renal clearance of the peptides but also extend their half-lives. Dose-dependent side effects such as nausea and gastrointestinal adverse events are the main drawbacks that are becoming more of a compliant with dose scaling.^28,29^ For instance, one newly approved GLP-1R agonist, semaglutide, has a noticeable half-life of 168 h thereby allowing weekly subcutaneous administration, while oral semaglutide (approved in 2019) formulated using absorption enhancer shows a similar half-life but is dosed daily with reported side effects (Table 2).^30,31^

**Table 2:** Newly approved drugs targeting class B GPCRs in the past 3 years

Drug	Brand name (manufacturer)	Indication	Target	GPCR class	FDA approval date
Erenumab-aooe	Aimovig (Amgen)	Migraine (prevention)	CALCRL	B1, peptide, calcitonin	05/17/2018
Ubrogepant	Ubrelvy (Allergan)	Migraine	CALCRL	B1, peptide, calcitonin	12/23/2019
Rimegepant	Nurtec ODT (Biohaven Pharm)	Migraine	CALCRL	B1, peptide, calcitonin	02/27/2020
Eptinezumab-jjmr	Vyepti (Lundbeck)	Migraine (prevention)	CALCRL	B1, peptide, calcitonin	02/21/2020
Semaglutide (injection)	Ozempic (Novo Nordisk)	Type 2 diabetes mellitus	GLP-1R	B1, peptide, glucagon-like peptide-1	12/05/2017
Semaglutide (oral)	Rybelsus (Novo Nordisk)	Type 2 diabetes mellitus	GLP-1R	B1, peptide, glucagon-like peptide-1	09/20/2019

The drugs listed above were identified manually from Drugs@FDA original NDA and BLA database (data extracted from August 2017 to June 2020) and cross-referenced with Drugbank,^20^ IUPHAR, and ChemBL databases

*CALCRL (CGRPR)* calcitonin gene-related peptide type 1 receptor

One of the latest approaches to develop more efficacious therapeutics against T2DM and obesity relates to dual- and tri-agonists targeting two or more of GLP-1R, glucagon receptor (GCGR), and glucose-dependent insulinotropic peptide receptor (GIPR). Many of them are currently in different phases of clinical trials (Table 3).^32–37^ Of note, in this receptor family, GLP-2 stimulates intestinal growth and an approved GLP-2R agonist, teduglutide, is used to treat short bowel syndrome.^38^

**Table 3:** Mono-, dual- and tri-agonists targeting GLP-1R, GCGR, and GIPR

Receptor	Drug	Dose form	Manufacturer	Status
GLP-1R mono-agonist	Exenatide	SC, twice daily	AstraZeneca	Approved
	Liraglutide	SC, once daily	Novo Nordisk	Approved
	Exenatide	SC, once weekly	AstraZeneca	Approved
	Lixisenatide	SC, once daily	Sanofi-Aventis	Approved
	Albiglutide	SC, once weekly	GlaxoSmithKline	Approved
	Dulaglutide	SC, once weekly	Eli Lilly	Approved
	Semaglutide	SC, once weekly	Novo Nordisk	Approved
	Semaglutide	Oral, once daily	Novo Nordisk	Approved
GLP-1R/GCGR dual-agonist	HM12525A	SC, once weekly	Hamni Pharmaceuticals	Phase 2
	JNJ54728518	SC	Janssen Pharmaceuticals	Phase 1
	MEDI0382	SC, once daily	MedImmune	Phase 2
	MK8521	SC, once daily	Merck	Phase 2
	NN9277	SC	Novo Nordisk	Phase 1
	MOD6030	SC, once monthly	Prolor Biotech, Opko Health	Phase 1
	SAR425899	SC, once daily	Sanofi-Aventis	Phase 2
	VPD107	SC, once weekly	Spitfire Pharma	Preclinical
	TT401	SC, once weekly	Transition Therapeutics	Phase 2
	ZP2929	SC, once daily	Zealand	Phase 1
GLP-1R/GIPR dual-agonist	CPD86	SC, once daily	Eli Lilly	Preclinical
	LY3298176	SC, once weekly	Eli Lilly	Phase 3
	NN9709/MAR709/RG7697	SC, once daily	Novo Nordisk/Marcadia	Phase 2
	SAR438335	—	Sanofi-Aventis	Trial discontinued
	ZP-I-98	SC, once weekly	Zealand	Preclinical
	ZP-DI-70	SC, once weekly	Zealand	Preclinical
GLP-1R/GCGR/GIPR tri-agonist	HM15211	SC	Hamni Pharmaceuticals	Phase 2
	MAR423	SC, once daily	Novo Nordisk/Marcadia	Phase 1

Data were retrieved from the literature^292,293^ and updated to Drugs@FDA, ChemBL, and ClinicalTrials.gov databases

*SC* subcutaneous

CGRP family has a considerable clinical relevance. For instance, pramlintide that targets amylin receptor is utilized to treat both type 1 and type 2 diabetes. Salmon calcitonin has been explored as a treatment for Paget’s disease and metabolic disorders.^39–41^ Furthermore, the association of migraine and CGRP elevation led to FDA-approved monoclonal antibodies (mAbs) against its receptor, e.g., erenumab and eptinezumab, as well as several small molecule antagonists such as rimegepant and ubrogepant (Table 2).^42,43^ Two approved diagnostic agents are analogs of CRF (corticorelin ovine triflutate peptide) and GHRH (sermorelin) for diagnosis of Cushing’s disease or ectopic adrenocorticotropic hormone syndrome and growth hormone deficiency, respectively.^44,45^ Tesamorelin, another synthetic form of GHRH, was approved in 2010 to treat human immunodeficiency virus (HIV)-associated lipodystrophy.^44^

PTH analogs, teriparatide and abaloparatide, were approved in 2002 and 2017, respectively, for postmenopausal osteoporosis with similar side effects. However, abaloparatide binds to parathyroid hormone 1 receptor (PTH1R) with higher affinity and selectivity that resulted in greater bone density.^46^

No therapeutic agent from the adhesion subfamily has entered clinical trial to date (Table S1).^2,4,47^ Although, adhesion GPCRs have shown coupling to heterotrimeric G proteins, the major challenge associated with this family is connecting G protein signals with biological activities.^24^ This subfamily was found to play functional roles in the immune, cardiovascular, respiratory, nervous, musculoskeletal, reproductive, renal, integumentary, sensory, endocrine, and gastrointestinal systems, with implications in neurological and neoplastic disorders.^24^ For instance, ADGRG1 and ADGRF1 are considered as potential drug targets due to their extensive pathogenetic involvement. Two ADGRG1/ADGRG5 modulators, dihydromunduletone and 3-α-acetoxydihydrodeoxygedunin developed via drug screening efforts, showed disease-related efficacy changes thereby calling for exploration of their activities in a pathological environment.^24,25^ However, associated drug resistance may not only hamper disease but also offer insights into potential mechanisms of such resistance and strategies to tackle it.

### Classes C and F

Class C (glutamate) contains 22 receptors, which are further divided into 5 subfamilies including 1 calcium-sensing receptor (CaSR), 2 gamma-aminobutyric acid (GABA) type B receptors (GABA_B1_ and GABA_B2_), 3 taste 1 receptors (TS1R1–3), 8 metabotropic glutamate receptors (mGluR1–8), and 8 orphan GPCRs.^48^ The distinctive features of glutamate subfamily are their large ECD and obligated constitutive dimer for receptor activation.^49^ The structural information of ECD indicates the roles of conserved venus fly trap (VFT) and cysteine-rich domain (CRD) on the ligand-binding site. Two conserved disulfide bonds between VFT domains stabilize the homodimers or heterodimers of class F GPCRs.^50^ The cryo-EM structures of the first full-length mGluR5^51^ and more recently the GABA_B_Rs further revealed their assembly mechanism and overall architecture.^52–55^ To date, 16 drugs have been approved by the FDA targeting 8 class C GPCRs. As archetypal receptors, mGluRs mediate the stimulus of agonists such as glutamate and their malfunction are implicated in various diseases, including cancer, schizophrenia, depression, and movement disorders. Acamprosate, an antagonist of mGluR5, was launched in 2004 as an anti-neoplastic agent.^56^ In fact, mGluRs have been vigorously pursued as therapeutic targets and there are 15 drug candidates undergoing clinical trials at present for pain, migraine, Parkinson’s disease, Fragile X syndrome, etc. Although allosteric modulators of class C have attracted significant development efforts involving 8 clinical trial stage compounds [2 positive (PAM) and 6 negative (NAM) allosteric modulators], the only success is cinacalcet, a small molecule PAM of CaSR approved in 2004 for hyperparathyroidism and calcimimetics.^57^

Only one class F GPCR (smoothed receptor SMO) has been validated as a drug target whose small molecule antagonists were approved as anti-neoplastic agents.^58^ Other 10 members of this class are all Frizzled receptors (FZD1–10), which mediate Wnt signaling and are essential for embryonic development and adult organisms. FZDs together with cognate Hedgehog and Wnt signal are associated with a variety of diseases such as cancer, fibrosis, and neurodegeneration.^59^ They share a conserved CRD in the extracellular part and ECD structures of SMO and FZD2/4/5/7/8 were determined.^60^ However, only SMO, FZD4, and FZD5 have TMD structures.^61–63^ Lack of full-length structures and complexity in signaling pathways impeded drug discovery initiatives.^60^ Linking of Wnt with extracellular CRD would activate downstream signaling, while the dimerization process and the interaction between CRD and TMD remain elusive.^64^ It is known that the downstream effectors of Wnt signaling consist of β-catenin, planar cell polarity, and Ca^2+^ pathways, whereas receptor activation involves in Wnt, Norrin, FZD, LDL receptor-related protein 5/6, and many other co-factors.^64^ Key breakthrough is thus required to advance our knowledge of these receptors.

## Medicinal chemistry of GPCR

### Agent type

**Fig. 2:**
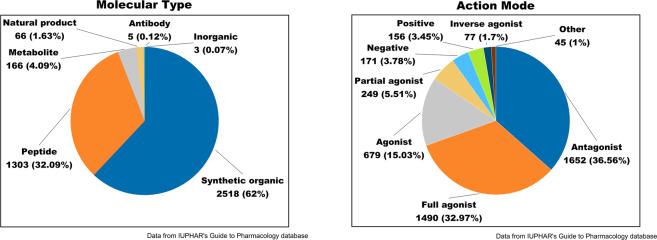
Analysis on agents targeting GPCRs. Distribution of molecule type (left) and action mode (right). Positive, PAM; Negative, NAM

Agents targeting GPCRs continue to expand in the past decades. Among them, exogenous small molecules, including traditionally developed synthetic organics, natural products, and inorganics, still dominate with a total percentage of 64% (Fig. 2). Nevertheless, the proportion of small molecules declines since 2010. In addition to traditional ligand discovery, several new modalities appear, though currently at the stage of academic research. Covalent ligands, with the embedding of reactive moieties that can be covalently linked to receptors, significantly enhance the weak binding of unoptimized leads.^65,66^ Photoactive ligands, developed by the introduction of photo-responsive groups to drug candidates, bring a new interdisciplinary field, photopharmacology. Albeit in its infancy, it has already found in vivo applications.^67,68^

In comparison, biologicals, such as peptides, antibodies, and metabolites, become more and more visible in the list. Particularly, the number of approved peptide drugs occupies approximately one third of the whole repertoire, with many more in different clinical stages as the pipeline^41,69^—most of them target classes A and B GPCRs. Naturally occurring peptides have been continually discovered from plants, animals, fungi, and bacteria. Although they act as efficient chemical messengers to modulate cellular functions, these peptides suffer from unfavorable pharmacokinetic and pharmacodynamics properties, such as very short plasma half-lives and low plasma protein binding. Therefore, chemical modifications are required to promote the membrane permeability, brain penetration, and oral bioavailability.^70^ Available strategies include peptide cyclization, *N*-methylation, palmitoylation, unnatural amino acid insertion, peptide–small molecule conjugation, and peptide self-assembly. By the way, developing peptidic agents may offer a new approach to de-orphanize certain orphan GPCRs.^71^

mAbs represent a promising alternative in GPCR drug discovery.^72,73^ Over small molecules, mAbs possess obvious advantages of improved specificity, affinity, and other pharmacological properties. Thus they are being developed against cancers, inflammation, and metabolic disorders. To date, three GPCR-targeting mAbs were approved (mogamulizumab, erenumab, and eptinezumab) while bi-specific antibodies, nanobodies, antibody–drug conjugates, and antibody–peptide conjugation are also in the development stage.

The emergence of many conceptually new molecular entities, such as RNA aptamer, provides not only powerful tool for biophysical study but also potential therapeutic candidates.^74^ Usually, aptamer has great molecular diversity and little immunogenicity.^75^ In addition, GPCRs are known to function by forming dimers (homodimers or heterodimers) and oligomers on the cell membrane.^76^ Therefore, strategies to induce receptor dimerization and/or oligomerization have received attention using scaffolds based on DNA (aptamer), small molecule, and physical stimuli.^77^

### Structure–activity relationship (SAR)

Studies of SARs are critical to the identification of drug-like molecules, especially when the crystal or cryo-EM structure of a drug target is not available. Given that many 3D GPCR structures have been solved in the past decade, most approved drugs were discovered without relevant structural information. Two examples are reviewed below to show the importance of SAR analysis.

**Fig. 3:**
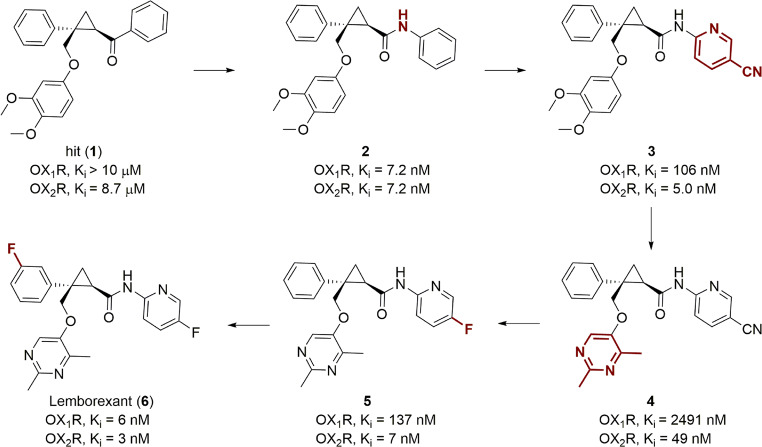
SAR studies that led to the discovery of the dual orexin receptor antagonist lemborexant

Orexin-1 and orexin-2 receptors (also known as hypocretin receptors, OX_1_R and OX_2_R) are class A GPCRs for which two endogenous peptide ligands were identified, orexin A and orexin B (also known as hypocretin 1 and hypocretin 2). The orexin signaling system plays a crucial role in regulating the sleep/wake cycle—both OX_1_R and OX_2_R are involved while the precise contribution of each has yet to be defined. Therefore, dual antagonists were developed as potential treatment for insomnia.^78^ Suvorexant (belsomra), the first-in-class dual orexin receptor antagonist, was launched in 2014.^79^ The second, lemborexant/E2006 (dayvigo) developed by Eisai, was approved by the FDA in 2020.^80^ It started from hit compound **1** (**6**, Fig. 3) with modest binding affinity to OX_2_R (*K*_i_ = 8.7 µM) and no affinity for OX_1_R.^81^ The first round of SAR studies revealed that changing the ketone group to an amide led to a remarkable enhancement (~1000-fold) of binding affinity at both OX_1_R and OX_2_R (compound **2**). Substitution of the aniline group with a 2-amino-5-cyano pyridine (compound **3**) maintained OX_2_R affinity and reduced OX_1_R activity, but physicochemical properties were improved compared to compound **2**.^81^ Further SAR studies focused on the modification of all three aromatic substitutions in compound **3**.^82^ Changing the *di*-OMe-phenyl substituent to a pyrimidine group resulted in a significant loss of binding affinity, as shown with compound **4**, but an improved overall profile due to reduced lipophilicity and enhanced solubility. Then replacing the cyano group to a fluorine regained the binding affinity for both receptors (compound **5**), and finally adding a second fluorine to the benzene group significantly improved OX_1_R affinity and led to lemborexant.^82^ Clearly, slight structural modifications may cause significant change of compound activity, and SAR studies coupled with optimization of physicochemical properties are useful steps to obtain druggable candidates.

**Fig. 4:**
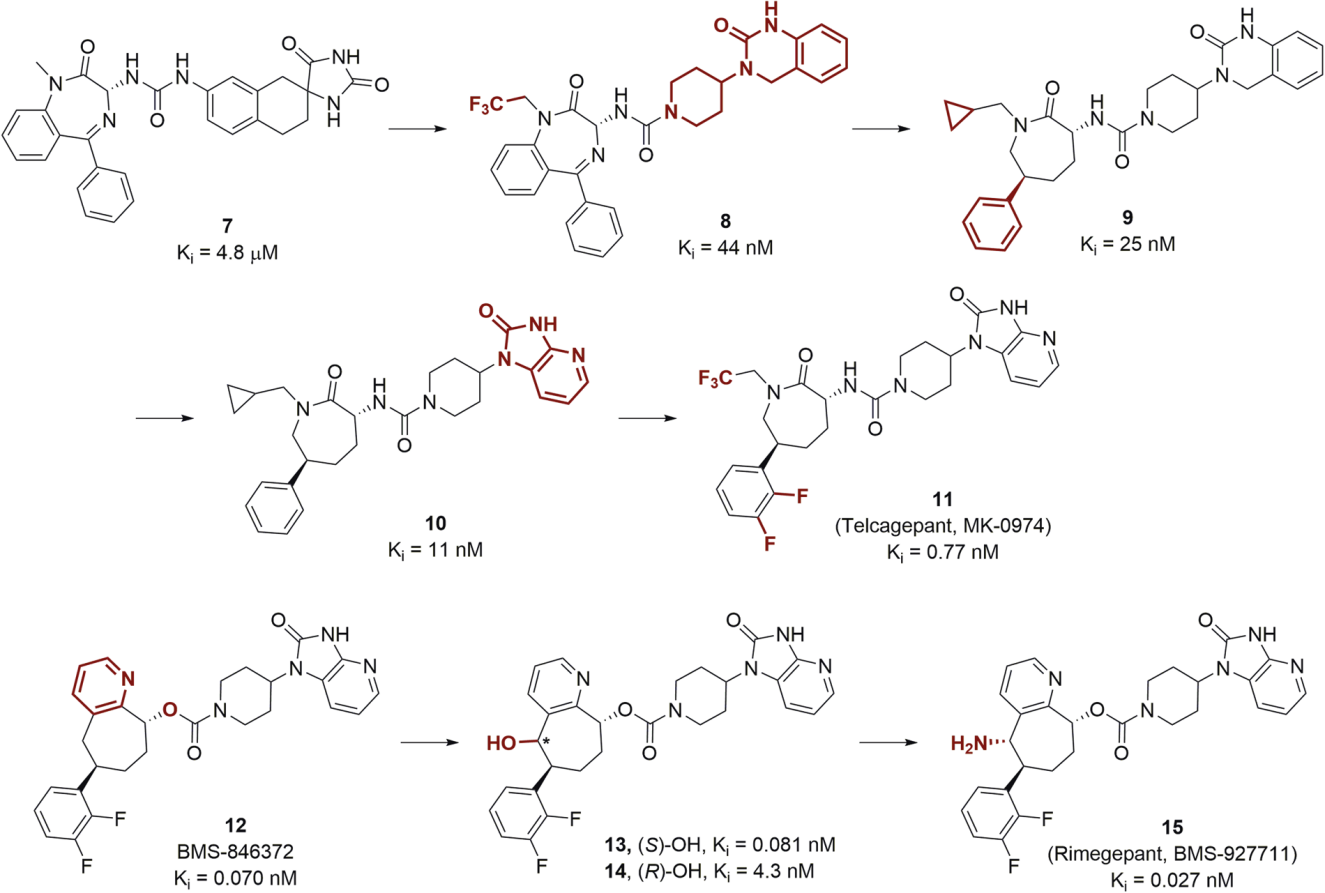
SAR studies that resulted in the discovery of CGRP antagonists

CGRP is a 37-amino acid neuropeptide and its receptor is implicated in migraine.^83^ The benzodiazapinone compound **7** was identified as a hit compound with modest CGRP receptor binding affinity (*K*_i_ = 4.8 µM, Fig. 4).^84^ Replacing the right-hand spirohydantoin structure with piperidyldihydroquinazolinone, a privileged structure for CGRP receptor antagonists,^85^ an affinity boost of 100-fold was gained.^84^ Further optimization of the benzodiazepinone core resulted in the caprolactam compound **9**, which showed a *K*_i_ of 25 nM.^86^ Changing the piperidyldihydroquinazolinone moiety to a piperidylazabenzimidazolone led to compound **10**, with a binding affinity of 11 nM.^87^ Then by changing the N-substituent on the caprolactam and adding *di*-fluro substitutions on the lower benzene ring delivered compound MK-0974 (**11**, *K*_i_ = 0.77 nM, Fig. 4), which entered clinical trials. Compound **12** (BMS-846372) shares the same piperidylazabenzimidazolone and the lower diflurobenzene substructures with **11** but differs from the latter with a carbamate core structure and a pyridine-fused-cyclopentane in replacement of the caprolactam.^88^ Compound **11** displayed high binding affinity while suffered from poor physicochemical properties, such as low solubility. To improve this, a hydroxyl group was attached to the cycloheptane ring and it was discovered that the (*S*)-isomer **13** was more potent than the (*R*)-OH compound **14**. The –OH was finally replaced with an -NH_2_ group, which led to the clinical compound rimegepant.^89^ The latter was further developed for better safety and efficacy profiles and obtained regulatory approval by the FDA in 2020.

The above examples demonstrate that, starting from a modest affinity hit compound, systematic SAR studies could successfully lead to very potent GPCR ligands that qualify as clinical candidates. Slight modifications of chemical structures sometimes cause remarkable changes of binding affinity or potency, which could not always be accurately predicted by conventional methods, such as docking. Therefore, SAR studies will continue to play a critical role in drug discovery.

## GPCR structure

The structure of GPCRs is a crucial determinant for understanding the molecular mechanisms underlying ligand recognition and receptor activation. It provides a foundation for drug discovery. The first crystal structure of inactive state rhodopsin purified from bovine eyes was solved in 2000.^90^ Although tremendous efforts have been made, elucidation of GPCR structures remains challenging due to several bottlenecks, including low receptor expression level, difficulties in extraction, highly flexible conformation, lack of crystal contacts, etc. The first crystal structure of GPCR extracted from exogenously expressed host cells, the human β2-adrenergic receptor (β_2_AR, gene name: *ADRB2*) bound to an antagonist, was disclosed in 2007, representing a milestone in GPCR structural biology.^7^ Several innovative methods, especially the incorporation of a soluble fusion partner and lipidic cubic phase (LCP) crystallization, facilitated subsequent studies. Further technological breakthroughs in protein expression and purification,^91,92^ receptor engineering,^8,93^ application of Fab fragment and nanobody,^94,95^ and GPCR crystallization^96^ led to an exponential growth of this field.

The crystal structure of β_2_AR in complex with stimulatory G protein (G_s_) solved in 2011^97^ and rhodopsin bound to visual arrestin reported in 2015^98^ revealed the molecular mechanism of GPCR interaction with G protein and arrestin, respectively. Notably, the wave of resolution revolution in the single-particle cryo-EM has brought a significant impact on the determination of GPCR complexes.^10^ Over 90% of GPCR–transducer complex structures were solved using cryo-EM (Table 4). To date, a total of 455 structures from 82 GPCRs belonging to all classes except B2 have been reported (Table 4). Although GPCRs show extensive sequence diversity, they share a conversed structural architecture of a TMD composed of seven helices embedded in the cell membrane. The transmembrane (TM) helices, essential for signal transduction across the cell membrane, are linked by three extracellular loops (ECLs) and three intracellular loops (ICLs). However, distinct structural features exist among members from different classes despite their overall structural similarity.

**Table 4:** List of GPCR structures

Receptor	Number of structures	PDB code (GPCR structure without downstream effector)	PDB code (GPCR structure with downstream effector)
*Class A*			
ADORA1	3	5N2S, 5UEN	**6D9H**
ADORA2A	49	2YDO, 2YDV, 3EML, 3PWH, 3QAK, 3REY, 3RFM, 3UZA, 3UZC, 3VG9, etc.	**6GDG**
ADRA2B	2		**6K41**, **6K42**
ADRB1	27	2VT4, 2Y00, 2YCW, 3ZPQ, 4AMI, 4BVN, 4GPO, 5A8E, 5F8U, 6H7J, etc.	**6TKO**, **7JJO**
ADRB2	33	2R4R, 2RH1, 3D4S, 3KJ6, 3NY8, 3P0G, 3PDS, 4GBR, 4LDE, 5D5A, etc.	3SN6, **6NI3**
AGTR1	6	4YAY, 4ZUD, 6DO1, 6OS1, 6OS2, 6OS0	
AGTR2	5	5UNF, 5UNG, 5UNH, 5XJM, 6JOD,	
APLNR	1	5VBL	
C5AR1	3	5O9H, 6C1Q, 6C1R	
CCR2	3	5T1A, 6GPS, 6GPX	
CCR5	6	4MBS, 5UIW, 6AKX, 6AKY, 6MEO, 6MET	
CCR6	1		**6WWZ**
CCR7	1	6QZH	
CCR9	1	5LWE	
CHRM1	3	5CXV, 6WJC	**6OIJ**
CHRM2	11	3UON, 4MQS, 4MQT, 5YC8, 5ZK3, 5ZK8, 5ZKB, 5ZKC	**6OIK**, **6U1N**, **6UP7**
CHRM3	5	4DAJ, 4U14, 4U15, 4U16, 5ZHP	
CHRM4	1	5DSG	
CHRM5	1	6OL9	
CNR1	7	5TGZ, 5U09, 5XR8, 5XRA, 6KQI	**6N4B**, **6KPG**
CNR2	4	5ZTY, **6KPC**	**6PT0**, **6KPF**
CXCR1	1	2LNL	
CXCR2	3	6LFL	**6LFM**, **6LFO**
CXCR4	6	3ODU, 3OE0, 3OE6, 3OE8, 3OE9, 4RWS	
CYSLTR1	2	6RZ4, 6RZ5	
CYSLTR2	4	6RZ6, 6RZ7, 6RZ8, 6RZ9	
DRD2	2	6CM4	**6VMS**
DRD3	1	3PBL	
DRD4	3	5WIU, 5WIV, 6IQL	
EDNRB	8	5GLH, 5GLI, 5X93, 5XPR, 6IGK, 6IGL, 6K1Q, 6LRY	
F2R	1	3VW7	
F2RL1	3	5NDD, 5NDZ, 5NJ6	
FFAR1	4	4PHU, 5KW2, 5TZR, 5TZY	
FFAR2	2	6LW5	**6OMM**
GPBAR1	2		**7CFM**, **7CFN**
GPR52	4	6LI1, 6LI2, 6LI0	**6LI3**
HCRTR1	11	4ZJ8, 4ZJC, 6TO7, 6TOD, 6TP3, 6TP4, 6TP6, 6TQ4, 6TQ6, 6TQ7, 6TQ9	
HCRTR2	6	4S0V, 5WQC, 5WS3, 6TPG, 6TPJ, 6TPN	
HRH1	1	3RZE	
HTR1B	4	4IAQ, 4IAR, 5V54	**6G79**
HTR2A	5	6A93, 6A94, 6WH4, 6WGT	**6WHA**
HTR2B	8	4IB4, 4NC3, 5TUD, 5TVN, 6DRX, 6DRY, 6DRZ, 6DS0	
HTR2C	2	6BQG, 6BQH	
LPAR1	3	4Z34, 4Z35, 4Z36	
MC4R	1	6W25	
MTNR1A	5	6ME2, 6ME3, 6ME4, 6ME5, 6PS8	
MTNR1B	4	6ME6, 6ME7, 6ME8, 6ME9	
NPY1R	2	5ZBH, 5ZBQ	
NTSR1	11	3ZEV, 4BUO, 4BV0, 4BWB, 4GRV, 4XEE, 4XES, 5T04	**6OS9**, **6OSA**, **6PWC**
OPRD1	4	4EJ4, 4N6H, 4RWA, 4RWD	
OPRK1	2	4DJH, 6B73	
OPRL1	3	4EA3, 5DHG, 5DHH	
OPRM1	4	4DKL, 5C1M	**6DDE**, **6DDF**
OXTR	1	6TPK	
P2RY1	2	4XNV, 4XNW	
P2RY12	3	4NTJ, 4PXZ, 4PY0	
PTAFR	2	5ZKP, 5ZKQ	
PTGDR2	2	6D26, 6D27	
PTGER3	2	6AK3, 6M9T	
PTGER4	2	5YHL, 5YWY	
RHO	55	1F88, 1GZM, 1HZX, 1L9H, 1LN6, 1U19, 2G87, 2HPY, 2I35, 2J4Y, etc.	4ZWJ, 5DGY, 5W0P, 6FUF, **6CMO**, **6OY9**, **6OYA**, **6QNO**
S1PR1	2	3V2W, 3V2Y	
TACR1	9	2KS9, 2KSA, 2KSB, 6HLL, 6HLO, 6HLP, 6J20, 6J21, 6E59	
TBXA2R	2	6IIU, 6IIV	
US28	4	4XT1, 4XT3, 5WB1, 5WB2	
*Class B*			
ADCYAP1R1	4		**6P9Y**, **6M1H**, **6M1L**, **6LPB**
CALCR	2		**5UZ7**, **6NIY**
CALCRL	4		**6E3Y**, **6UVA**, **6UUN**, **6UUS**
CRHR1	4	4K5Y, 4Z9G	**6PB0**, **6P9X**
CRHR2	1		**6PB1**
GCGR	9	4L6R, 5EE7, 5XEZ, 5XF1, 5YQZ	**6LMK**, **6LML**, **6WHC**, **6WPW**
GHRHR	1		**7CZ5**
GLP-1R	12	5NX2, 5VEW, 5VEX, 6KJV, 6KK1, 6KK7, 6LN2	**5VAI**, **6B3J**, **6ORV**, **7C2E**, **6VCB**
GLP-2R	1		**7D68**
PTH1R	4	6FJ3	**6NBF**, **6NBH**, **6NBI**
SCTR	2		**6WZG**, **6WI9**
VIPR1	1		**6VN7**
*Class C*			
GABBR2	8	**7C7S**, **7C7Q**, **6UO8**, **6VJM**, **6UOA**, **6UO9**, **6W2X**, **6WIV**	
GRM1	1	4OR2	
GRM5	8	4OO9, 5CGC, 5CGD, 6FFH, 6FFI, 6N4X, **6N51**, **6N52**	
*Class F*			
FZD4	1	6BD4	
FZD5	1	**6WW2**	
SMO	11	4JKV, 4N4W, 4O9R, 4QIM, 4QIN, 5L7D, 5L7I, 5V56, 5V57, 6O3C	**6OT0**

The structures were updated in September 2020. The PDB codes of GPCR structures determined by cryo-EM are in bold. The structural data were collected from the Protein Data Bank (rcsb.org)^294^

*ADCYAP1R1* pituitary adenylate cyclase-activating polypeptide type I receptor, *ADORA1 (A*_*1*_*AR)* adenosine A1 receptor, *ADRA2B* α2B adrenergic receptor, *ADRB1 (β*_*1*_*AR)* β1-adrenergic receptor, *AGTR2 (AT2R)* angiotensin II receptor type 2, *APLNR* Apelin receptor, *C5AR1* C5a anaphylatoxin chemotactic receptor 1, *CALCR (CTR)* calcitonin receptor, *CCR1–9* C-C chemokine receptor (CCR) type 1–9, *CHRM2 (M2R)* muscarinic acetylcholine receptor M2, *CHRM3 (M3R)* muscarinic acetylcholine receptor M3, *CHRM4 (M4R)* muscarinic acetylcholine receptor M4, *CNR2 (CB2)* cannabinoid receptor 2, *CRHR1 (CRF1R)* corticotropin-releasing factor receptor 1, *CRHR2 (CRF2R)* corticotropin-releasing factor receptor 2, *CXCR1–2* C-X-C chemokine receptor type 1–2, *CYSLTR1-2* cysteinyl leukotriene receptor 1–2, *DRD4* D4 dopamine receptor, *EDNRB* endothelin receptor type B, *F2R* proteinase-activated receptor 1, *F2RL1* proteinase-activated receptor 2, *FFAR1-2* free fatty acid receptor 1-2, *GABBR2 (GABA*_*B2*_*)* GABA type B receptor subunit 2, *GHRHR*^295^ growth hormone-releasing hormone receptor, *GLP-2R*^296^ glucagon-like peptide-2 receptor, *GPBAR1* protein-coupled bile acid receptor, *GPR52* G protein-coupled receptor 52, *HCRTR2 (OX*_*2*_*R)* orexin receptor type 2, *HRH1* histamine H1 receptor, *HTR1B* 5-hydroxytryptamine receptor 1B, *HTR2B (5-HT*_*2B*_*)* 5-hydroxytryptamine receptor 2B, *HTR2C (5-HT*_*2C*_*)* 5-hydroxytryptamine receptor 2C, *LPAR1* lysophosphatidic acid receptor 1, *MC4R* melanocortin-4 receptor, *MTNR1A* melatonin receptor type 1A, *MTNR1B* melatonin receptor type 1B, *NPY1R* neuropeptide Y receptor Y1, *NTSR1* neurotensin receptor type 1, *OPRD1* delta-type opioid receptor, *OPRL1*, nociceptin receptor, *OPRK1 (κ-OR)* kappa-type opioid receptor, *OPRM1 (µ-OR)* mu-type opioid receptor, *OXTR* oxytocin receptor, *P2RY1* P2Y purinoceptor 1, *P2RY12* P2Y purinoceptor 12, *PTAFR* platelet-activating factor receptor, *PTGDR2* prostaglandin D2 receptor 2, *PTGER3* prostaglandin E2 receptor EP3 subtype, *PTGER4* prostaglandin E2 receptor EP4 subtype, *RHO* rhodopsin, *SCTR* secretin receptor *SMO* smoothened homolog, *TBXA2R* thromboxane A2 receptor, *US28* G-protein coupled receptor homolog US28, *VIPR1* vasoactive intestinal peptide receptor 1

**Fig. 5:**
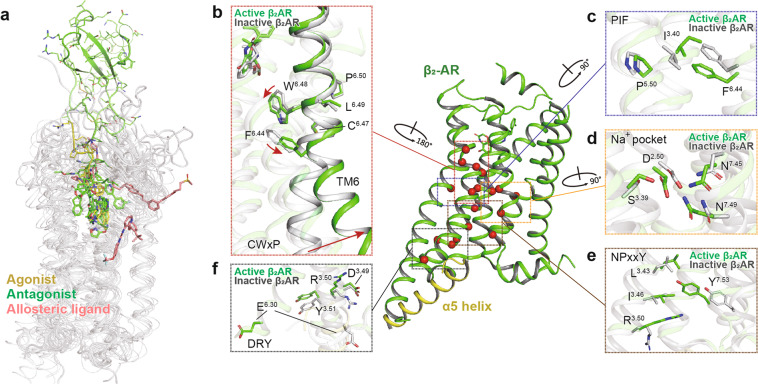
Structural features and common activation mechanism of class A GPCRs. **a** Ligand-binding pockets. Agonist, antagonist, and allosteric ligand are indicated as sticks in yellow, green, and salmon, respectively. Ligands are shown from the following structures (PDB code): 2RH1, 3PWH, 3VW7, 4IAR, 4MQT, 4PHU, 4RWS, 4XEE, 4XNV, 4Z35, and 4ZJ8. **b**–**f** The common activation pathway of class A GPCRs as exampled by the structures of inactive (gray, PDB code 3NYA) and active β_2_AR (green, PDB code 3SN6). The conformational changes of conserved “micro-switches”, including CWxP (**b**), PIF (**c**), Na^+^ pocket (**d**), NPxxY (**e**), and DRY (**f**), are highlighted. Side chains of residues in “micro-switches” are shown as sticks. Red arrows indicate the shift and swing directions of elements in the active β_2_AR structure relative to the inactive one

Various ligands of class A GPCRs bind to similar orthosteric sites directly in the helix bundle. Structural variations in ECLs, TM helices, and side chains show a remarkable variety of sizes, shapes, and physicochemical properties of the ligand-binding pockets, leading to diversified mechanisms of ligand recognition.^99^ For example, ligand binding, access, and selectivity are affected by ECL2.^100,101^ Many published GPCR structures are in an antagonist-bound inactive state, but the number of agonist-bound active state structures have been increased steadily in recent years due to the deployment of cryo-EM. Additionally, the structure of human M2R bound to a PAM (LY2119620) unveiled the allosteric ligand recognition mechanism.^102^ A summary of complicated recognition and modulation mechanisms of class A GPCRs bound to agonist, antagonist, and PAM is illustrated in Fig. 5a.

Class A GPCRs are activated through a common pathway, which strings the conserved “micro-switches” together, including CWxP, PIF, Na^+^ pocket, NPxxY, and DRY, thereby linking the ligand-binding pocket to the G protein-coupling region (Fig. 5b–f).^99,103^ The binding of diverse agonists triggers the rotameric switch of W^6.48^, a highly conserved residue in the “CWxP” motif, and the concomitant side chain rotation of F^6.44^ (Fig. 5b). Upon stimulation by an agonist, conformational rearrangement occurs in the PIF (P^5.50^, I^3.40^, and F^6.44^, Fig. 5c) and the Na^+^ pocket residues (D^2.50^, S^3.39^, N^7.45^, and N^7.49^, Fig. 5d). These reorganizations rigger the notable outward displacement of TM6, the hallmark of class A GPCR activation (Fig. 5b). The repacking of Na^+^ pocket residues initiates the TM7 movement toward TM3. Upon receptor activation, the “NPxxY” residue Y^7.53^ changes its rotamer conformation and points toward TM3, rendering new contact formation between Y^7.53^ and residues in TM3 (L^3.43^, I^3.46^, and R^3.50^, Fig. 5e) and subsequently the enhanced packing of TM3–TM7. Finally, “DRY”, one of the most conserved motifs in class A receptors, locates at the bottom of the 7TM and forms an intra-helical salt bridge between D/E^3.49^ and R^3.50^. R^3.50^ forms an additional inter-helical salt bridge with D^6.30^, known as the ionic lock, which connects the intracellular ends of TM3 and TM6 to stabilize receptors in an inactive state (Fig. 5f). These contacts are eliminated after agonist binding, and R^3.50^ is released to interact with other residues to facilitate the G protein coupling. It is notable that an acidic residue at position 6.30 is less conserved in 30% of class A receptors. Alternatively, R^3.50^ may form polar interactions with other polar residues in TM6 (i.e., T^6.34^ in κ-OR and μ-OR) to mediate the activation. Collectively, these rearrangements and reorganizations of conserved motifs are critical to the activation of class A GPCRs.

**Fig. 6:**
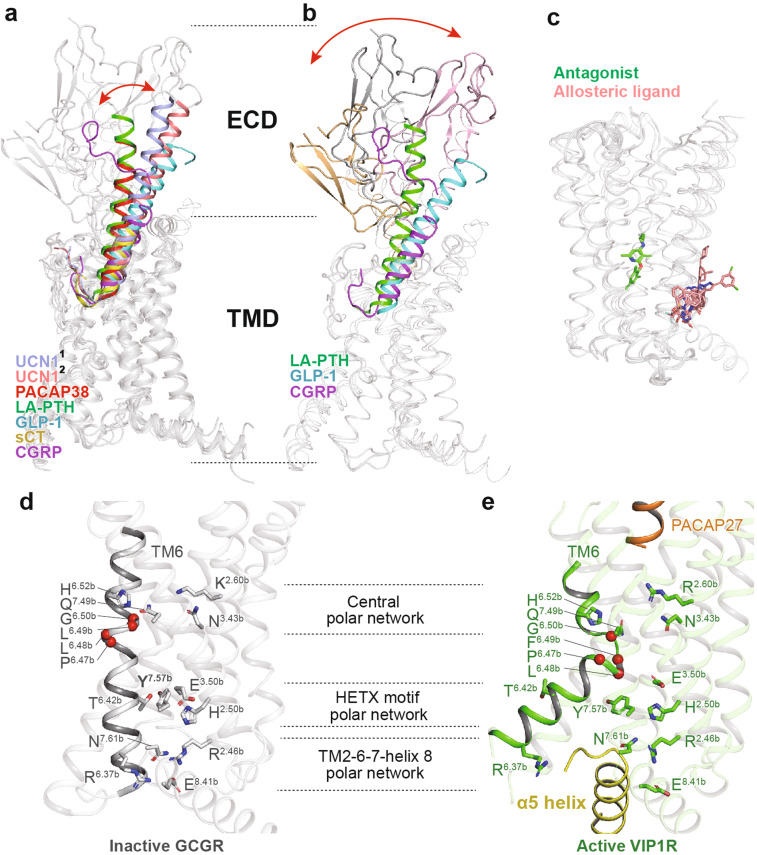
Structural features and common activation mechanism of class B GPCRs. **a**, **b** Structural features of the peptide-binding pocket. The shift of peptide C-terminus (**a**) and ECD (**b**) is indicated as red arrows. The peptides urocortin 1 (UCN1)^1^ bound to CRF1R (light blue, PDB code: 6PB0), UCN1^2^ bound to CRF2R (salmon, PDB code: 6PB1), PACAP38 (red, PDB code: 6P9Y), long-acting PTH (LA-PTH, green, PDB code: 6NBF), GLP-1 (cyan, PDB code: 5VAI), sCT (yellow, PDB code: 6NIY), and CGRP (magenta, PDB code: 6PB1) are shown as cartoons. Binding poses of the antagonist (green) and allosteric ligand (salmon) are shown as sticks (**c**, PDB codes: 4K5Y, 5EE7, 4Z9G, 5VEW, and 5VEX). **d**, **e** The common activation mechanism of class B GPCRs as exampled by the structures of inactive GCGR (gray, PDB code 3NYA) and active VIP1R (green, PDB code 6VN7). Side chains of residues in three conserved polar network are shown in stick presentation. The conserved P^6.47b^xxG^6.50b^ motifs in TM6 are shown as single red spheres

Class B GPCRs contain a large ECD and a TMD bundle with the peptide ligand recognition by both domains. According to the two-domain-binding model, the C-terminus of the peptide interacts with the ECD and orient the N-terminus of the peptide toward the TMD bundle. It then engages with the TMD core to facilitate receptor activation.^104^ The most remarkable structural feature of this class is the swing of ECD, accompanied by the corresponding shift of the peptide C-terminus (Fig. 6a, b). Conversely, the N-terminus inserts into a V-shape cavity within the helix bundle with a similar binding pose. Compared to small molecule-binding pocket of class A, that of class B is more solvent-accessible with higher flexibility and larger volume to accommodate sizeable peptidic ligands.^9^ In addition, structural studies also reveal an antagonist-binding pocket deep in the TMD bundle of CRF1R^105^ and a common binding site for allosteric modulators of GCGR^106^ and GLP-1R^107^ located outside the TMD bundle between TM6 and TM7 (Fig. 6c).

A comparison of the full-length active receptor structures with that in the inactive state reveals a general activation mechanism for class B GPCRs (Fig. 6d, e).^9,108^ The binding of a peptidic ligand causes a conformation rearrangement of the central polar network with simultaneous destabilization of the TM6 helix, thus initiating a sharp kink formation at the conserved motif P^6.47b^xxG^6.50b^. This central polar network is preserved across the class B receptors solved so far. However, the exact interactions may vary among different members in a ligand- and receptor-specific manner. The rearrangement of TM6 breaks the polar interaction of the conserved HETX motif and the TM2-6-7-helix 8 polar network, thereby inducing a notable outward displacement of TM6 and creating a cytoplasmic cavity to accommodate α5 helix of Gα_s_ protein.

**Fig. 7:**
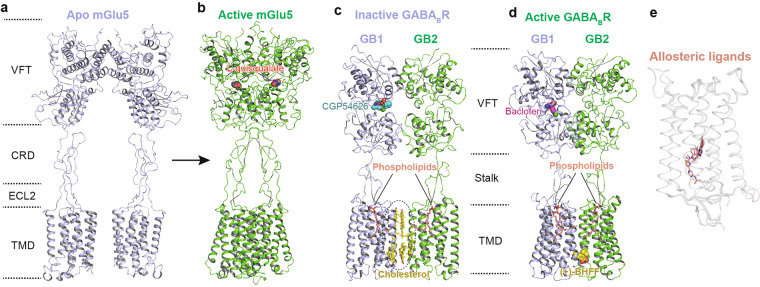
Structural features and activation mechanism of class C GPCRs. The structures of mGlu5 in resting state (**a**, PDB code: 6N52) and active state (**b**, PDB code: 6N51), as well as GABA_B_R in inactive (**c**, PDB code: 7C7S) and active states (**d**, PDB code: 7C7Q) are displayed, respectively. Agonists L-quisqualate (**b**, magenta) and antagonist CGP54626 (**c**, cyan) of mGlu5 as well as agonist baclofen (**d**, magenta) and allosteric modulator (+)-BHFF (**d**, yellow) of GABA_B_R are shown as spheres. Cholesterols (**c**, yellow) and phospholipids (**c**, **d**, salmon) are indicated as sticks. Binding of allosteric ligands to TMD of class C GPCR is indicated as salmon sticks (**e**, PDB codes: 4OR2, 4OO9, 5CGC, and 6FFH)

Class C GPCRs are distinguished by a characteristically large ECD that forms an obligate dimer. The ECD is distal to the TMD and contains an orthosteric ligand-binding pocket. It is composed of a ligand-binding VFT linked by the CRD to the TMD except for the metabotropic GABA_B_ receptor (GABA_B_R), which lacks CRD (Fig. 7a–d). This structural feature results in a potentially unique ligand recognition mechanism. The full-length structures of mGlu5 in *apo* and agonist-bound states,^51^ as well as several recently reported full-length structures of GABA_B_Rs,^52,53,55^ have significantly extended our understanding of the activation mechanism of the class C receptors. It is known that an agonist binds and stabilizes the conformation of the VFT, leading to compaction of the inter-subunit dimer interface and proximity of the CRD (Fig. 7a, b). This conformation transition, in turn, triggers TMD rearrangement through interaction between ECL2 and CRD.^51–53,55^ In contrast to mGlu5, the GABA_B_R undergoes a featured asymmetric activation. After the binding of agonist baclofen to GABA_B1_ (GB1) subunit, the latter only exhibits a negligible conformational change. Additionally, due to lacking CRD in the GABA_B_Rs, the relatively shorter stalk and ECL2 region may rigidify their conformations and mediate the transduction of conformational changes from VFT to 7TM.^53^ In contrast, substantial conformational alterations occur at the stalk and TM3/4/5-ICL3 regions at the cytoplasmic part of GB2 (Fig. 7c, d), which predominantly couples to G_i1_ heterotrimer. Interestingly, cholesterols are observed at the TMD interface of inactive GABA_B_Rs^52^ (Fig. 7c), while two chained phospholipids occupy a binding site overlapped with the orthosteric binding pocket in class A GPCRs^52,53^ (Fig. 7c, d). These cholesterols and phospholipids may contribute to the activity regulation of the GABA_B_R. Noteworthy, in contrast to other allosteric modulators that bind to the TMD core of class C GPCRs (Fig. 7e),^109–111^ (+)-BHFF occupies a novel allosteric site at the interface of TMDs in GB1 and GB2 subunits^52^ (Fig. 7d). This novel allosteric binding site may provide a promising template for the design of PAMs for GABA_B_Rs.

**Fig. 8:**
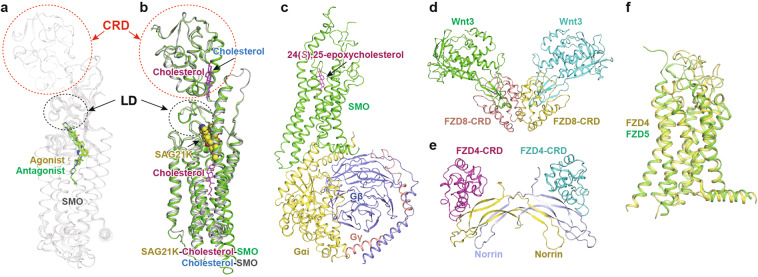
Structural feature of class F GPCRs. **a** Superposition of SMO crystal structures bound to agonists (yellow sticks) and antagonists (green sticks). The following structures are shown (PDB codes): 4JKV, 4N4W, 4O9R, 4QIM, and 5V56. CRD and LD (linker domain) are highlighted; **b** A comparison of structures of full-length SMO in the active state (PDB codes: 5L7D and 6O3C). Cholesterols are indicated. SAG21K, the agonist of SMO, is shown as yellow spheres. **c** The cryo-EM structure of SMO TMD in complex with G_i_ heterotrimer (PDB code: 6OT0). The agonist 24(S),25-epoxycholesterol is shown as magenta sticks. **d** Crystal structures of the Wnt3-FZD8 CRD complex. **e** Crystal structures of the Norrin-FZD4 CRD complex. **f** A comparison of the *apo* TMD structures of FZD4 (PDB code: 6BD4, yellow) and FZD5 (PDB code: 6WW2, green)

Class F GPCRs include SMO and 10 FZDs in humans. Besides a canonical TMD across all classes of GPCRs, class F is characterized by a large ECD composed of a CRD and an ECD linker domain to connect with TMD (Fig. 8a, b).^112^ It was reported that SMO has a unique allosteric modulation mechanism.^113^ In fact, two ligand-binding sites have been identified: one in CRD and the other in TMD (Fig. 8b). SMO is activated by cholesterol via binding to CRD. The binding of an antagonist to TMD was proposed to trigger its conformation change thereby propagating to CRD and allosterically impeding the binding of cholesterol.^113^ Recent structural studies reveal that cholesterol and oxysterol that are critical for SMO activation are located deep within the 7TM domain of SMO (Fig. 8b, c).^114,115^ CRD of FZD can interact with lipoglycoprotein Wnt and Norrin (specific ligand for FZD4) to mediate the Wnt signaling.^61,116^ Structures of CRD in complex with Wnt or Norrin provided molecular details of how they formed a symmetrical homodimer (2:2 complex) during ligand recognition (Fig. 8d, e).^117,118^ In contrast to SMO, the ligand recognition and receptor activation mechanisms of FZD remains elusive due to the absence of the full-length FZD structures. So far, only two *apo* TMD structures of FZD4 and FZD5 have been reported (Fig. 8f).^61,63^ Structures of the full-length FZD in a ligand-bound state are required awaiting to provide mechanistic explanations.

## GPCR pharmacology

The explosion of 3D GPCR structures and computational simulations has revealed the dynamic conformations between inactive, intermediate, and active states of GPCRs. The detailed structural information illustrated that cholesterol, ion, lipids, and water also participate in receptor activation.^99,119^ The flexibility of receptor-binding pocket endows the complex pharmacological mechanisms of ligand recognition and signal transduction. Biased signaling, allosteric modulation, and polypharmacology are helping us better understand how GPCRs bind to numerous ligands and how they transmit diverse signals to elicit physiological functions.

### Polypharmacology

Ligand binding to multiple targets leads to antagonism, additive, or synergism pharmacological responses that could be positive or negative based on the mechanism of action. The paradigm of one drug vs. multiple targets has outpaced the time and cost associated with the conventional therapy.^120^ Polypharmacology thus emerges to study acceptable degree of specificity toward multiple targets, interconnected signaling pathways that result in clinical benefit or cross-reactivity that may cause adverse events.^121,122^ T2DM, obesity, cancer, and Alzheimer’s disease are major indications for GPCR modulators.^4^ These polygenic diseases are not completely treatable by a single agent, while desirable efficacies may be achieved for certain respiratory conditions, central nervous system (CNS) disorders, and cardiovascular diseases through modulators directed against β_2_AR, DRD2, and AGTR1,^123^ respectively.

It was shown that 5-hydroxytryptamine receptor 2 (5-HT_2_) binds to selective inverse (ritanserin) and highly promiscuous (ergotamine) agonists but the interaction with ergotamine is broad.^121^ This feature allows the development of pan serotonin receptor modulators to treat different diseases.^124–127^ For instance, zolmitriptan as an anti-migraine drug is also used for hyperesthesia via binding to off-target site,^120^ and lorcaserin (Belviq) is used to treat obesity while its therapeutic potential for depression, schizophrenia, and drug addiction is being investigated.^128,129^ However, off-target activity, hallucinations,^130^ and cardiac valvulopathy related to 5-HT_2A_ and 5-HT_2B_ modulation^129^ should be carefully monitored. Atypical antipsychotics are mainly targeting both dopamine and serotonin receptors, usually as antagonist for DRD2 and antagonist or inverse agonist for 5-HT_2A._^131^ Exemplified by clozapine^120^ and aripiprazole,^132^ haloperidol, amoxapine, and asenapine^4^ display a diverse spectrum of receptor interaction. Additionally, carazolol, a member of aminergic division exerts its effects by interacting with multiple adrenergic receptors as inverse agonist or allosteric antagonist.^19,129^ Istradefylline combined with L-DOPA/dopamine simultaneously target A_2A_R, DRD1 and DRD2 in animal model of Parkinson’s disease.^131^ Amitryptyline, a tricyclic compound targeting muscarinic and histamine H1 receptors,^133^ is used to treat depression and non-selective muscarinic receptor antagonists are trialed for bladder dysfunction.^4^ Lorazepam, indicated for anxiety due to interaction with GABA_A_R, is also an allosteric modulator of the proton-sensing GPCR (GPR68)^134^ and has been repurposed to treat pancreatic cancer.^2^ 6’-Guanidinonaltrindole (6’-GNTI) is an agonist with higher selectivity for δ/κ-opioid receptor heterodimer but not homodimer. Importantly, 6’-GNTI is an analgesic that offers additional benefit. In cardiovascular diseases, β blockers decrease catecholamine-induced heart rate elevation via interaction with valsartan (AT1R-mediated signaling).^135^ It is of note that mono-, dual-, and tri-agonists for the glucagon family of receptors (GLP-1R, GCGR, and GIPR) have been developed and trialed for weight loss and glucose control (Table 3). Successful outcome will determine whether unimolecular polypharmacology is a practical approach to translate safety and efficacy of multiple agents into a single molecule.^136^

### Biased agonism

Activated GPCRs can recruit multiple transducers (such as heterotrimeric G proteins, GPCR kinases, and β-arrestin) and consequently produce distinct biological responses. Ligands that preferentially engage one signaling pathway over others are regarded as bias and may show improved therapeutic outcomes.^137,138^ Biased signaling that has been applied to drug discovery involve AT2R, µ-OR, κ-OR, β-adrenergic receptors, DRD2, CTR, CCR, and adenosine receptors. µ-OR is the best studied receptor for biased agonism.^137^ Compounds that stimulate Gα_i_ coupling and cAMP production but not β-arrestin recruitment are preferable to retain analgesia and reduce opioid-related side effects.^139^ This G protein bias was also demonstrated with widely used drug tramadol,^140^ whose active metabolite, desmetramadol, elicited maximum cAMP production without affecting β-arrestin 2 recruitment compared to fentanyl and morphine. Safety profile is improved with less adverse effect such as respiratory depression.^140^ Another µ-OR-biased ligand, oliceridine (TRV130, Olinvo^TM^), passed phase III clinical trial but did not get the FDA approval for safety concerns.^141^ The NDA for oliceridine was resubmitted and a new counterpart, TRV734, is not only suitable for oral administration but also safer due to reduced dependency.^142^ A fourth µ-OR-biased ligand, PZM21, cross-reacts with κ-OR and failed to reduce respiratory depression in C57BL and CD-1 mice.^143^ Whether this relates to its residual but marked effect on β-arrestin 2 recruitment, as opposed to oliceridine whose action is negligible,^144^ remains to be further studied.

Similar situation occurred with κ-OR as well whose agonists possess analgesic property and have a low risk of dependence and abuse but with adverse effects such as sedation, motor dysfunction, hallucination, and dysphoria.^145^ G protein-biased agonists of κ-OR,^146^ including RB-64,^145^ mesyl salvinorin B, triazole 1.1, diphenethylamines and LOR17,^141^ were reported to minimize the adverse effects in preclinical settings. One of such, nalfurafine, was approved in Japan (2015) as an anti-pruritic agent for patients with chronic liver diseases.^147^

Carvedilol, known as a β1 and β2 adrenoceptor blocker, was found to be biased toward β-arrestin recruitment, G protein-coupled receptor kinase activation, and ERK1/2 phosphorylation. Joining its rank included alprenolol, bucindolol, and nebivolol, all are used to treat hypertension and congestive heart failure.^148^ In the case of β3 adrenoceptor, CL316243 is cAMP-biased, whereas L748337 and SR59230 are ERK/p38 phosphorylation-biased.^149,150^ Interestingly, CL316243 was also tested for treatment of obese mice.^151,152^ However, none of them have advanced to the clinic.

In contrast to µ-OR, arrestin bias is desirable for AT1R to improve cardiac performance.^153^ Nonetheless, clinical development of AT1R modulators either resulted in a phase IIb trial failure (TRV027) in 2017^154^ or never reached to clinical stage (SBpa, SVdF, SI, sarmesin, saralasin, and SII).^155^ Of note is that biased molecules may show species preference. For instance, CL316243 is more active in mice than in humans,^156^ whereas nalfurafine works better in humans vs. rodents.^157^ A list of therapeutic agents with biased signaling approved or advanced to clinical trials is shown in Table 5.

**Table 5:** Therapeutic agents with biased signaling approved or in clinical trials

Ligand	Receptor (GPCR class)	Signaling bias	Indication	Development status	Reference
Bromocriptine	Serotonin receptors, adenosine receptors, dopamine receptors (class A)	At 5HT_2_ G_q/11_; at DRD2 β-arrestin	Acromegaly, Parkinson’s disease, T2DM, idiopathic hyperprolactinemic disorder, neuroleptic malignant syndrome	Approved	^297–299^
Pergolide	5HT_2_ (class A)	G_q/11_	Parkinson’s disease	Approved	^297,298^
Ergotamine	HTR2B (class A)	β-arrestin	Migraine	Approved	^297,300^
Atropine	CHRM3 (class A)	Low efficacy agonist for G_12_, inverse agonist for Gq, antagonist for G_i_	Organophosphorous poison antidote	Approved	^297,301^
Pilocarpine	CHRM3 (class A)	β-arrestin and pERK1/2	Xerostomia	Approved	^297,302^
Capadenoson	ADRA1A (class A)	cAMP	Atrial fibrillation	Phase 2	^297,303^
Alprenolol, bucindolol, carvedilol, nebivolol	ADRB1 and ADRB2 (class A)	β-arrestin	Congestive heart failure	Approved	^148,304^
Isoetharine	ADRB (class A)	β-arrestin	Asthma	Approved	^304,305^
Dihydrexidine (DAR-0100A)	DRD2 (class A)	Full agonists for G_s_ but partial agonists for G_olf_ signaling	Schizotypal personality disorder	Phase 2	^297,306^
Bifeprunox	DRD2 (class A)	Kinetic bias	Bipolar disorder, depression, schizophrenia, psychosis	Phase 3	^297,307^
Aripiprazole	DRD2 (class A)	Kinetic bias	Psychosis	Approved	^297,307^
TRV027 (TRV120027)	AGTR1 (class A)	β-arrestin	Anti-hypertensive with cardio-protection	Phase 2	^5,304^
TRV250	OPRD1 (class A)	G protein	Migraine	Phase 1	^5,137^
Nalfurafine	OPRK1 (class A)	G protein	Pruritus	Approved	^157^
Tramadol	OPRM1 (class A)	G protein	Pain	Approved	^140^
Oliceridine (TRV130)	OPRM1 (class A)	G protein	Pain	Phase 3	^5,137^
TRV734	OPRM1 (class A)	G protein	Pain	Phase 1	^5,137^
CYT-1010	OPRM1 (class A)	G protein	Pain	Phase 1	^308^
Satavaptan (SR121463)	AVPR2 (class A)	β-arrestin (partial agonist) while inverse agonist at G_s_	Hyponatremia and ascites	Phase 3	^297,309^
Atosiban	OXTR (class A)	G_i1_ and G_i3_	Delaying imminent preterm birth	Approved	^297,310^
BMS-986104	S1PR1 (class A)	cAMP	Rheumatoid arthritis	Phase 1	^297,311^
LY2828360	CNR2 (class A)	G_i_/ERK	Knee osteoarthritis	Phase 2	^297,312^
MK-0354	HCAR2 (class A)	G protein	Dyslipidemia	Phase 2	^297,313^
TRV027 (TRV120027)	AGTR1 (class A)	β-arrestin	Anti-hypertensive with cardio-protection	Phase 2	^5,304^
Exenatide	GLP-1R (class B)	β-arrestin	T2DM	Approved	^12^
TTP273	GLP-1R (class B)	G protein	T2DM	Phase 2	^190^

Receptor abbreviations are according to IUPHAR. The list is derived from earlier reports^5,137,304,308^ with addition based on literature research. The indication and development status are updated from DrugBank.ca and ClinicalTrials.gov database

*ADRA1A* alpha-1A adrenergic receptor, *AVPR2 (V2R)* arginine vasopressin receptor 2, *HCAR2* hydroxycarboxylic acid receptor 2

### Allosteric modulation

In recent years, studies on allosteric GPCR modulators have gained unprecedented momentum.^158–161^ An allosteric modulator is a ligand binding to a position other than the orthosteric site but can modify responses of a receptor to stimulus. Allosteric modulators that enhance agonist-mediated response are called PAMs, while those attenuate the response are called NAMs. This phenomenon is very common such that the Allosteric Database 2019 (ASD, http://mdl.shsmu.edu.cn/ASD)^162^ records 37520 allosteric modulations on 118 GPCR members, covering all four classes.

Allosteric modulation is advantageous in terms of (i) using highly druggable pockets. In some cases, it is easier to design ligands at an allosteric site than the orthosteric site, such as class B GPCRs with orthosteric pockets wide open. For example, both PAM^163^ and NAMs^107^ binding to the same position at the TMD of GLP-1R were reported; (ii) improving selectivity. The orthosteric site and cognate ligand are often highly conserved, making it hard to discover very selective orthosteric binders. Meanwhile, non-conserved allosteric sites would be a better choice evidenced by discovery of many subtype selective allosteric modulators of acetylcholine^102,164^ and cannabinoid receptors^165,166^; (iii) introducing signal bias. Allosteric modulators with biased signaling were developed for prostaglandin F2α receptor^167^ and chemokine receptor CXCR4.^168^ Albeit still as an emerging concept, allosteric modulators have exhibited a great potential with some compounds being marketed or in clinical trials.^160^

However, developing allosteric modulators of GPCRs remains challenging—molecules recorded in the ASD largely concentrate on two subfamilies, the mGluRs (8 members, 17,115 modulations), and mAChRs (5 members, 7666 modulations), accounting for nearly 2/3 of the total number. Some individual receptors also contribute a significant proportion, such as CB1 (1948 modulations), GABA_B_ (1286 modulations), and follicle-stimulating hormone receptor (1233 modulations). Excluding these “easy cases,” allosteric modulators are few in number. Furthermore, the structural diversity of the allosteric modulators is quite low, for many derivatives would be included soon after a parent compound is identified. The difficulty in developing allosteric modulators is partly due to the limitation of detecting allosteric behavior: Not every newly discovered active compound could be tested for its effect on binding affinity or EC_50_ of an orthosteric agonist, therefore some allosteric modulators were not correctly identified. For instance, BPTU in P2RY1, the first GPCR NAM solved in complex structure (PDB code: 4XNV),^169^ was not considered allosteric until the structure was obtained. To make things worse, NAMs may weaken the binding of an endogenous ligand thus behaving like a competitor, such as NDT9513727 in C5AR1^170^ (PDB code: 5O9H).^171^

**Fig. 9:**
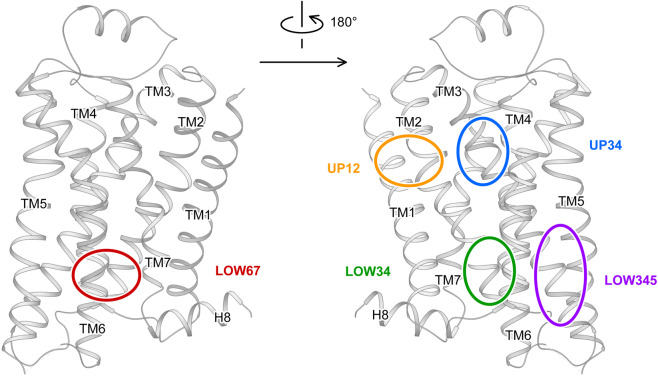
Schematic diagram of allosteric sites at the lipidic surface identified by complex structures. The binding sites are manually labeled on the crystal structure of β_2_AR (PDB code: 6OBA^180^). Solid line, allosteric site at front side; dashed line, allosteric site at back side. UP, upper part aka close to the extracellular end; LOW, lower part aka close to the cytoplasmic end; numbers, main interacting transmembrane helices

**Fig. 10:**
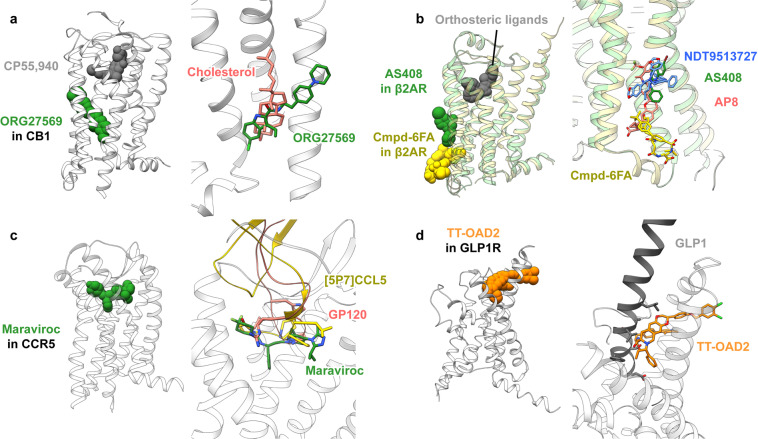
Binding sites of allosteric modulators in GPCRs reported after October 2018, in comparison with related ligands. **a** NAM ORG27569 in CB1 (PDB code: 6KQI^166^) in comparison with cholesterol (PDB code: 5XRA^179^); **b** NAM AS408 (PDB code: 6OBA^180^) and PAM Cmpd-6FA (PDB code: 6N48^181^) in β_2_AR, in comparison with NDT9513727 in C5AR1 (PDB code: 6C1Q^182^) and PAM AP8 (PDB code: 5TZY^183^); **c** NAM maraviroc in CCR5 (PDB code: 4MBS^187^) in comparison with chemokine analog antagonist [5P7]CCL5 (PDB code: 5UIW^188^) and HIV envelope glycoprotein gp120 (PDB code: 6MEO^189^); **d** PAM TT-OAD2 in GLP-1R (PDB code: 6ORV^190^) in comparison with GLP-1 (PDB code: 5VAI^191^)

The most effective way to identify the binding site of an allosteric modulator on a GPCR is solving the complex structure. Crystallography is an effective technique, while rapidly deployment of cryo-EM has started to deliver its promise (PDB codes: 6OIK^172^ and 6U1N^173^). To date, 17 GPCRs have reported structures in complex with allosteric modulators. Detailed analysis of complex structures before October 2018 was reported previously,^161^ and here we focus on insights provided by newly published results. The most unusual allosteric-binding sites on GPCRs are at the lipidic interface embedded in cell membrane. Five different positions were identified by crystal structures (Fig. 9): UP12, UP34, LOW34, LOW345, and LOW67. Four of them were recently reviewed.^161^ The LOW34 site was reported in 2019 for ORG27569 in CB1 (PDB code: 6KQI^166^; Fig. 10a).

ORG27569 attracted much attention for its distinctive function: increasing the binding of orthosteric agonist CP55940 but making it act as inverse agonist.^165^ Many attempts were made to locate the binding site of ORG27569 by mutagenesis but the results are conflicting: one study showed that the effect of ORG27569 on CP55940-induced [^35^S]GTPγS binding was disturbed by mutations to multiple residues at the orthosteric site, leading to a hypothesis that ORG27569 stays in the same pocket close to CP55940.^174^ Another study found that ORG27569 reduced the binding of a fluorescence-labeled orthosteric antagonist, and the effect was only disturbed by mutations at the lipidic interface close to the cytoplasmic end of CB1.^175^ Besides, it was reported that the functions of ORG27569 were also affected by breaking a disulfide bond at the N-terminus^176^ or by constitutive active/inactive mutations at the cytoplasmic interface.^174,175,177,178^ The crystal structure exhibited that the position of ORG27569 is considerably overlapped with a cholesterol captured in another intermediate state (PDB code: 5XRA,^179^ Fig. 10a), consistent with the site located by the fluorescence-labeled orthosteric antagonist.^175^ At this site, the higher selectivity to CB1 over CB2 could be explained. Interestingly, ORG27569 is the only allosteric modulator at lipidic interface forming no hydrogen bond to the receptor.

There have been three more complex structures of allosteric modulators at lipidic interface since October 2018, all obtained by crystallography. Two are β_2_AR, with a NAM AS408 (PDB code: 6OBA^180^) or a PAM Cmpd-6FA (PDB code: 6N48^181^). Both allosteric modulators bind to the LOW345 site (Fig. 10b). The NAM stays at a position very similar to NAMs in C5AR1 (PDB codes: 5O9H,^171^ 6C1R, and 6C1Q^182^) but the PAM is close to ICL2 and only partially overlaps with PAMs of FFAR1 (PDB codes: 5TZY^183^ and 5KW2^184^), showing a complex regulation nature at this site. The other complex structure is full-length GLP-1R with PF-06372222 (PDB code: 6LN2^185^), a NAM previously used to co-crystallized with GLP-1R TMD (PDB code: 5VEW^107^).

Even around the position of orthosteric ligands (among the helices and facing extracellular side), another ligand may occupy the space not taken by the endogenous ligand and act as an allosteric modulator. The very abundant PAMs/NAMs of mAChRs function in this mechanism. PAM LY2119620 in M2R (with the orthosteric agonist iperoxo and stabilized by a nanobody) was the first allosteric modulator to obtain complex structure with a class A GPCR (PDB code: 6MQT^102^). Recently, LY2119620 was also observed in protein complexes of M2R with G protein (PDB code: 6OIK^172^) or arrestin (PDB code: 6U1N^173^) by cryo-EM.

CCR5 is a chemokine receptor and an important anti-HIV drug target. A marketed inhibitor, maraviroc, has long been recognized as a NAM of CCR5. There were hypotheses that small molecule NAMs, chemokine, and the HIV-binding protein have separate binding sites.^186^ However, structures of CCR5 in complex with maraviroc (PDB code: 4MBS^187^), chemokine analog antagonist (PDB code: 5UIW^188^), or HIV envelope glycoprotein (PDB code: 6MEO^189^) show that these ligands highly overlap in CCR5 pocket (Fig. 10c). Therefore, the noncompetitive behavior of maraviroc may be due to a very extensive interface of peptidic CCR5 agonist, thus a small molecule cannot diminish the binding even with this much collision. The results illustrate that allosteric behavior is not equal to totally separated binding positions, because partially overlapped sites with different key interactions are also allowed.

The last case of allosteric modulator in extracellular pocket is PAM TT-OAD2 of GLP-1R (PDB code: 6ORV^190^). This small molecule agonist only slightly collides with the endogenous peptide (PDB code: 5VAI^191^, Fig. 10d), consistent with its behavior that only partially displaces an orthosteric probe.^190^

The cytoplasmic interface, where a GPCR interacts with intracellular partners, including Gα and β-arrestin, contains pockets suitable for drug design. So far, four small molecules have been validated by crystallography to bind at this position. The targets are three chemokine receptors (CCR2, PDB code: 5T1A^192^; CCR7, PDB code: 6QZH^193^; and CCR9, PDB code: 5LWE^194^) and β_2_AR (PDB code: 5X7D^195^). These ligands are all NAMs and proximately share the same binding site (TM1, TM2, TM6, TM7, ICL1, and H8). Their binding position does not overlap with Gα, therefore they may stabilize the inactive state by blocking conformational changes required for receptor activation. This site is generally non-conserved in the GPCR superfamily, thus targeting here may provide some selectivity. Additionally, many nanobodies at the cytoplasmic interface were also developed for several receptors, including AGTR1 (PDB codes: 6DO1^196^ and 6OS0^197^), β_1_AR (PDB code: 6IBL^198^), β_2_AR (PDB code: 6N48^181^), and SMO (PDB code: 6O3C^114^; for information before October 2018, see review^161^).

Multi-domain regulation is an interesting topic in allosteric modulator discovery. Class C GPCRs use ECDs to recognize their cognate ligands, leaving the classic pocket of TMD for allosteric modulating.^161,199^ This is the major reason why this class has a large number of allosteric modulators. In the case of mGluRs, both PAMs and NAMs have been widely reported, but only NAMs obtained complex structures—there is no solved active state structure. The full-length structures of mGlu5 (PDB codes: 6N51 and 6N52^51^) displayed how the binding of orthosteric agonist to ECD triggers the change of interaction between two monomers, but the conformational change of TMD remains elusive.

SMO in class F is also a multi-domain receptor. The first reported ligand of SMO cyclopamine (an antagonist causing birth defects) binds to the classic TMD pocket (PDB code: 4O9R^200^) shared by several other antagonists with different chemical scaffolds and an agonist (SAG).^62,113,201,202^ ALLO-1, an antagonist identified as allosteric modulator not competitive to cyclopamine, was recently found to bind at a deeper position in the pocket by photo-affinity labeling combined with mass spectrometry (MS).^203^ SMO has another pocket in ECD that interacts with steroids, including cholesterol (PDB codes: 5L7D^113^ and 6D35^204^) and 20(*S*)-hydroxycholesterol (PDB code: 5KZV^119^). Since cholesterol has been the most favored candidate of SMO endogenous ligand, the ECD pocket is treated as orthosteric making the TMD pocket allosteric. However, newly obtained structures demonstrated that cholesterol or its analog can also bind to TMD pocket (PDB codes: 6O3C^114^ and 6OT0^115^), leaving the question open for which is the true orthosteric site.

### Disease indication

GPCRs are involved in many human diseases and specific drug intervention is one of the most celebrating achievements in the pharmaceutical industry (Table S3 and Fig. S1). Among all available drugs targeting GPCRs, HRH1, DRD2, M1R, and ADRA1A are the most frequently addressed for indications such as hypertension, allergy, pain, and schizophrenia, and 33% of them have >1 indication with an overall average of 1.5. Although CNS diseases are still popular accounting for 26% of all approved indications, development focuses have now been shifted to T2DM, obesity, multiple sclerosis, smoking cessation, short bowel syndrome, and hypocalcemia. Repurposing of existing drugs for new indications also emerged to supplement discovery efforts.

## Structure-based drug design

As two general types of computer-aided drug design techniques (Table 6),^205^ SBDD and ligand-based drug design, exploit the structural information of protein targets and the knowledge of known ligands, respectively (Table 6). SBDD, on the basis of crystal/cryo-EM/NMR structures or homology models, first identifies key sites and important interactions responsible for target functions, then screens large virtual library/designed agents that disrupt or enhance such interactions to modulate relevant biological processes and/or signaling pathways by molecular docking, and finally discovers active leads with desired pharmacological properties.

Clearly, the past decade is a golden age for SBDD on GPCR. With the year of 2011 (when LPC crystallization,^206^ fusion proteins,^8^ and other key techniques collaborated to launch the outbreak of GPCR structure determination including the landmark β_2_AR-G_s_) for watershed,^207^ SBDD of GPCR evolves two distinct stages: rhodopsin-based homology model and truly authentic structure of individual receptors. Boosted by the fast-increasing number of high-quality GPCR structures, improved accuracy of combinational computational approaches, and better understanding of activation mechanism and pharmacology, SBDD is developing rapidly with fruitful scientific reports and increasing GPCR-targeted drugs contributed by this approach. Considering the length of time required for a drug to be available on the market (10–15 years) and the chance of applying structural biology information to hit discovery and lead optimization in the first 2–3 years of a drug discovery program, it is probably too early to see the approval of GPCR-targeted drugs being developed with the aid of a structure, and such situation is likely to change as the tremendous efforts from both academia and industry start to bear the fruits of successes. The following is a brief account of recent advances in three main aspects of SBDD (chemical space, receptor dynamics, and pose evaluation) in the context of their application in GPCR pharmacology.

### Optimized virtual library

Despite the vast chemical space (>10^63^ drug-like molecules), only a nominal fraction has been explored by SBDD, where both the compound availability and insufficient diversity limited the number of screened ligands. To overcome these problems, ultra-large^208–210^ and focused libraries^211–213^ were employed. Lyu and colleagues^208^ presented an excellent model of “bigger is better” in virtual drug screening. Based on the 130 well-characterized reactions, they generated 170 million make-on-demand compounds (http://zinc15.docking.org/), the resulting library is remarkably diverse with >10.7 million scaffolds unavailable before. By docking 138 million molecules against DRD4, they discovered 81 new chemotypes (24% hit rate), 30 of them showed submicromolar activity, including a 180-pM subtype-selective and G_i_-biased DRD4 agonist. This ultra-large library docking study provides important information: (i) hit rate fell almost monotonically with docking score; (ii) hit rate vs. score curve of DRD4 predicted that 1 from every 873 compounds may have a minimum affinity of 1 μM; and (iii) human visual evaluation improved the selected compound with higher affinities, efficacies, and potencies but not the hit rate. A follow-up study on MT_1_ by docking >150 million “lead-like” molecules^209^ identified 15 active leads (39% hit rate) with potencies ranging from 470 pM to 6 μM. Alternatively, focused library^210–215^ including scaffold library, natural products, dark chemical matter (i.e., chemicals that have never shown bioactivity tested in over 100 assays), and fragment- and lead-like libraries were introduced in virtual screening (VS) for dozens of receptors. Focused on compound library of traditional Chinese medicine (TCM), Liu et al. found that salvianolic acids A and C antagonized the activity of both P2RY1 and P2RY12 purinoceptors in the low µM range, while salvianolic acid B antagonized the P2RY12 purinoceptor. Remarkably, these three salvianolic acids are major active components of the broadly used hemorheologic TCM Danshen (*Salvia militorrhiza*). Taking NTSR1 as an example, Ranganathan et al. found that the fragment library tended to have higher hit rate than that of the lead-like library (19%) but the affinities were ∼100-fold weaker. Collectively, these results demonstrate the importance and advantages of ultra-large and tailored libraries in discovering potent GPCR modulators.

### Receptor dynamics

Emerging evidence from crystallography, spectroscopy, and molecular dynamics (MD) simulations have demonstrated the crucial roles of GPCR dynamics involved in ligand recognition, receptor activation, and allosteric modulation.^216–218^ To consider the protein flexibility during GPCR-related SBDD, many computational approaches^217^ including rotamer sampling, induced-fit docking, and ensemble docking have been employed showing a great promise, especially in the search of biased, bi-topic, or allosteric modulators. During ensemble docking,^212,217,219–221^ ligands are docked into multiple structures representing different possible conformational states rather than a single structure, where the targets could be multiple crystal structures or extracted from MD/Monte Carlo (MC) simulations or normal mode analysis (NMA). By evaluating the known ligand enrichment, as well as selectivity for agonists or antagonists on seven GPCR/ligand co-structures, Coudrat et al. found that small variations in structural features are responsible for their success in VS, while a combination of ligand/receptor interaction patterns and predicted interaction strength is associated with the predictive power of binding pockets in VS.^220^ Compared to the Glide VS workflow, the combination of accelerated MD simulations and Glide induced fit docking of M2R by Miao et al. provided much-improved enrichment factors and identified four PAMs and one NAM with unprecedented chemical diversity.^221^ For 5-HT_1A_ whose crystal structure is not available, Warszycki et al. applied MC and NMA to generate an ensemble of binding pockets with the input of a homology template and known active compounds and finally discovered two new active ligands through VS.^222^

### Pose evaluation

Correctly selecting and ranking poses of docked compounds in the ligand-binding pockets have been a challenge for SBDD, especially for GPCR that is embedded in the cell membrane with significant conformational adaptability. To address this problem, many physics-based scoring functions^223–226^ integrated with some user-friendly computer programs (e.g., Dock, GOLD, AutoDock, Glide, and rDock) were routinely adopted in SBDD. Recently, precise computational approaches including free energy calculation methods like molecular mechanics/Poisson–Boltzmann surface area (MM/PB(GB)SA),^227,228^ free energy perturbation (FEP),^229^ quantum mechanical/MM calculations,^230^ and fragment molecular orbital^231,232^ have been employed with improved performance. Compared to the empirical scoring functions, MM/PB(GB)SA and FEP are physically more rigorous free-energy calculation methods with an increased computational cost and have been adopted in the studies of DNA–ligand, protein–ligand, and protein–protein interactions.^233,234^ By introducing of the minimization-based MM/GBSA refining and rescoring of docked poses, Zhou et al. identified seven 5-HT_2B_ antagonists with novel chemical scaffolds and the most potent one has an IC_50_ of 27.3 nM in a cellular assay.^227^ Lenselink et al. used FEP to design A_2A_R antagonists and identified a highly potent molecule with *K*_i_ of 1.2 nM.^232^ However, computational investigation across 20 class A crystal structures and 934 known ligands demonstrated that the correlations between predicted binding free energy by MM/PBSA and experimental data varied significantly. The observed variations exist between individual receptors and are highly system specific,^235^ indicating that successful application of MM/PBSA may require additional efforts in validation of experimental data and optimization of simulation/calculation parameters. Alternatively, protein–ligand interaction fingerprints exacted from available crystal structures^225,236^ fueled docking score with protein–ligand-binding mode information and resulted in improved VS virtual hit rates for β_2_AR (53%) and HRH1 (73%) with up to nM affinities and potencies.^225,236^

Collectively, innovation in VS and pose evaluation, along with evolution of computational hardware, has significantly advanced SBDD and is expected to lift the discovery efficiency to a new height, since GPCRs have multiple downstream signaling pathways responsible for distinct functions or consequences. The high degree of sequence and pocket similarities between different subtypes demands for novel ligands with superior specificity and selectivity. In this regard, allosteric and biased modulators may offer additional pharmacological benefits.

### Subtype selectivity

It is known that GPCR subtypes share high sequence identities in orthosteric sites with distinct distribution and downstream signaling profiles. Cross-reactivity among subtypes could cause undesired side effects. For example, five MR subtypes display different G protein coupling features (G_q/11_: M1R, M3R and M5R; G_i/o_: M2R and M4R) and organ distribution (CNS, M1R; peripheral tissues such as heart and colon, M2R), while their sequence identify (64–82%) and similarity (82–92%) in TMD are quite conserved.^172^ Similar observations were seen among dopamine receptors (DRD1 to DRD5), histamine receptors (HRH1 to HRH4), and adenosine receptors (A_1_AR, A_2A_R, A_2B_R, and A_3_AR). Guided by structural information, rational design of subtype selective compounds progresses steadily. Using an extended DRD2-specific binding pocket from the haloperidol-bound DRD2 crystal structure, Fan et al. discovered two highly selective DRD2 agonists (O4SE6 and O8LE6) that specifically activate DRD2 (EC_50_ = 1 µM) after screening of 320 non-olfactory GPCRs.^237^ Through VS of 3.1 million molecules against M2 and M3, Kruse et al. identified a partial M3 agonist without measurable M2 agonism, capable of stimulating insulin release from a mouse β-cell line.^238^ Wei et al. reported a multistage VS of the ChemDiv library (1,492,362 compounds) toward A_1_AR and discovered four novel antagonists with good affinity and selectively (>100-fold) over A_2A_R.^239^

### Biased signaling

Recently, Suomivuori et al. performed extensive MD simulations to identify two major signaling conformations that couple effectively to arrestin or G protein, respectively. They then designed ligands via minor chemical modification resulting in strong arrestin-biased or G_q_-biased signal transduction.^240^ Meanwhile, McCorvy and colleagues discovered that specific ECL2–ligand contacts are associated with β-arrestin recruitment, whereas blockage of TM5 interaction reduces the G_i/o_ signaling. An arrestin-biased DRD2 modulator was thus made exhibiting a calculated bias factor of 20 relative to quinpirole.^241^ In addition, Mannel et al. conducted a VS of a tailored virtual library bearing 2,3-dichlorophenylpiperazine for DRD2 and found that 18 compounds occupy both orthosteric and allosteric sites, and 4 of them stimulated β-arrestin recruitment (EC_50_ = 320 nM, *E*_max_ = 16%) without detectable G protein signaling.^214^

### Allosterism

In the past 5 years, an increasing number of receptor–allosteric modulator complex structures revealed diversified positions of allosteric sites and a variety of binding modes, thereby deepening our understanding of allosteric modulation in terms of underlying mechanisms and structural basis. Further to conventional orthosteric ligands, allosteric modulators affect receptor function in different ways. While PAM may enhance maximal efficacy, NAM could reduce agonist signaling strength.^159,160,242^ It was reported that a PAM of M2R is located above the orthosteric site and interacts with ECLs. Korczynska et al. screened 4.6 million molecules against the allosteric sites of M2R and identified a PAM that potentiated the action of antagonist *N*-methyl scopolamine (NMS). Subsequent optimization led to a subtype-selective compound **628** that increased NMS binding with a co-operativity factor of 5.5 and a *K*_B_ of 1.1 µM.^16^ Alternatively, Lückmann et al. carried out MD simulations of agonist-removed FFAR1 and found that closure of a potential allosteric site is associated with agonist binding—compounds that bind to this site to prevent the closure functions as allosteric agonists.^243^ Obviously, aided by >400 structures from 82 receptors, SBDD is now entering into a new era with substantial knowledge of GPCR signaling^103,110,244^ and drug candidate attributes.^245^

## Novel screening technology

As GPCRs represent the most prominent family of therapeutic targets,^132^ innumerable efforts have been made in both industry and academia to screen for novel ligands that can modulate the activity of a specified GPCR and serve as lead compounds for drug development.

A diverse array of experimental technologies suitable for assaying protein–ligand interactions have been directly applied or tailored to GPCR-targeted ligand screening, and they can be classified into three main categories: binding-based, stability-based, and cell signaling-based assays (Table 7). Binding-based assays monitor the physical interactions between a GPCR protein typically in a purified recombinant form with individual test compounds. Cell signaling-based assays measure downstream effectors (e.g., cAMP, Ca^2+^, IP1/IP3) of specific intracellular signaling pathways known to be mediated by GPCR, which reflect the functional outcome of ligand binding to the receptor. Stability-based assays assess the variation of thermal stability for a purified protein when treated by test compounds. These different techniques vary in the ligand screening throughput and binding characteristics (Table 7). In the lead discovery stage, both binding- and signaling/activity-based assays are implemented in a parallel or sequential manner, as the multipronged use of complementary techniques would reduce the overall false-positive and false-negative rates.^246^

**Table 6:** Comparison of the advantages and disadvantages of various computer-aided drug design approaches

	Approach	Advantage	Disadvantage
Ligand-based drug design (LBDD)	Quantitative structure–activity relationship^314,315^ (QSAR)	Understanding interactions between functional groups, convenient, does not require the structural information of a target	Descriptor selection, false correlations, enough known ligands
	Pharmacophore modeling^316,317^	Effective model, convenient, does not require the structural information of a target	Known ligands, less novelty; missing conformations
Structure-based drug design (SBDD)	Virtual screening of ultra-large libraries^224–226^	Novel scaffolds and chemotypes, higher chances of finding potent ligands	Substantial computational resources, compound synthesis
	Virtual screening of focused libraries^227–229^	Less demand for computational resources, compound easy to purchase, specific scaffolds or origins	Reduced diversity, less novelty
	Ensemble docking^235–237^	Considering protein flexibility, improved enrichment factors, rescue of false-negative ligands	Increased computational burden, pose evaluation, false positive
	Energy-based pose evaluation^248–252^	Improved scoring and ranking ability	Substantial computational burden, method validation target dependency

Here we summarize about 20 experimental screening technologies adapted to GPCR ligand discovery (Table 7) and highlight the most recent development of binding-based approaches. Notably, an update of assays assessing GPCR activation and signaling has been provided in a previous review^247^ and will not be elaborated here. The structure-based VS is covered in the above section. Structural elucidation technologies (e.g., X-ray crystallography, single-particle cryo-EM, NMR, and HDX-MS) not suitable for high-throughput screening (HTS) are also excluded.

### DNA-encoded library (DEL)

Impressive technological advances have been made for binding-based ligand screening over the past decade. Specifically, DEL has emerged as a powerful approach to drug discovery.^248–251^ Created by split and pool synthesis, DEL usually contains hundreds of thousands to billions of distinct small molecule–DNA conjugates. A majority of DEL-based HTS reported to date involve incubation of an immobilized target protein with the library before the protein–ligand complexes are isolated. Encoding DNA tags associated with the immobilized target are then amplified and sequenced to assign relevant chemical structures.^250,251^ Although DEL was predominantly applied to ligand screening against soluble proteins such as enzymes, successful adaptation of this technique to GPCRs was reported in a few cases.^252–255^ Lefkowitz group reported the discovery of a NAM for β_2_AR by screening a DEL of 190 million.^252^ This NAM not only has a unique chemotype but also exhibits low μM affinity and inhibits cAMP production as well as β-arrestin recruitment. Later on, the same team discovered the first small molecule PAM for β_2_AR through HTS of >500 million DEL compounds.^253^ Both NAM and PAM demonstrated high selectivity. Of note is that the NAM was found using unliganded β_2_AR, whereas the PAM was unmasked via intentional application of β_2_AR with its orthosteric site occupied by an agonist thereby shifting the receptor to the active state.^252,253^ These two studies elegantly demonstrated a proof-of-concept strategy for binding-based screening of allosteric modulators targeting different conformational states.^253^

The power of DEL in the discovery of allosteric GPCR modulators was further demonstrated for PAR2.^254^ Screening a billion-size library with a thermostabilized PAR2 mutant resulted in the identification of several agonists and antagonists, and some of them bind to an allosteric pocket in the TMD of PAR2. A similar approach was used to discover tachykinin receptor neurokinin-3 (NK3) antagonists of low nM potency involving NK3 overexpressing cells and a library containing tens of millions DNA-encoded compounds.^255^ Clearly, DEL-based ligand screening against GPCRs and other integral membrane proteins offers great promises as it circumvents difficulties in receptor purification.

### Affinity selection MS

Due to the high sensitivity and high selectivity of modern MS for both protein and small molecule analysis, versatile MS-based technologies have been developed in the past two decades for screening ligands of a given protein target or characterization of ligand-binding properties (Table 7). Almost all of them were originally developed for measuring ligand interactions with soluble proteins,^256–261^ and recently they have been adapted to more challenging GPCR drug discovery. The majority of MS-based technologies (e.g., automated ligand identification system (ALIS), ultrafiltration–liquid chromatography/MS, frontal affinity chromatography–MS, membrane-based affinity MS, and competitive MS binding) employ a methodology very similar to DEL as they all capture and detect ligands physically associated with a given GPCR,^262–267^ except that native MS analyzes the entire ligand-bound receptor complexes.^268,269^ In general, these methods have several advantages over ligand binding or cell signaling assays: (i) unbiased and direct detection of ligand–receptor binding facilitates the identification of both orthosteric and allosteric modulators; (ii) confirmation of ligand identity with accurate mass measurement; (iii) no chemical labeling or DNA encoding of test compounds; and (iv) quantitative MS analysis enables ranking of ligand affinity or evaluation of binding characteristics.

**Table 7:** Diverse GPCR ligand screening technologies classified into three categories

Category	Method	Assay principle	Readout	Activity characterization	Throughput
Binding-based assay	Radiolabeled ligand binding	Detect binding of a radioisotope-labeled ligand to a target in competition with a test compound	Radioactivity	*K*_i_, *K*_on_, *K*_off_	Medium
	DEL	DNA-encoded compounds bound to a target are affinity selected and their structures revealed by DNA sequencing	DNA sequence	Affinity ranking	Ultra-high
	SPR	Detect changes in the refractive index of the gold film surface when ligands interact with a target immobilized on the chip surface	Refractive index (mass on surface)	*K*_d_, stoichiometry (*n*), *K*_on_, *K*_off_	Medium
	MST	Detect directed movement of molecules through a temperature gradient using covalently attached or intrinsic fluorophores	Fluorescence intensity	*K*_d_, stoichiometry (*n*)	Medium
	TR-FRET	Detect fluorescence resonance energy transfer caused by interaction between target and ligand labeled with specific fluorophores	Fluorescence intensity	*K*_i_	Medium
	ALIS	Target–ligand complexes are isolated by fast SEC, and dissociated ligands are identified by high-res MS	*m*/*z* and MS intensity	Affinity ranking, *K*_d_, ACE_50_	High
	Membrane-based affinity MS	Target–ligand complexes in the cell membrane are separated from solution by filtration prior to ligand identification by high-res MS	*m*/*z* and MS intensity	Affinity ranking	High
	UF-LC/MS	Target–ligand complexes are separated from solution by ultrafiltration prior to ligand identification by LC-MS	*m*/*z* and MS intensity	Affinity ranking, *K*_d_	High
	FAC-MS	Detect ligands flowing through a protein-immobilized column based on breakthrough curves determined by MS	*m*/*z* and MS intensity	Affinity ranking, *K*_d_	Medium
	Competitive MS binding	Detect binding of a non-radioactive ligand to a target in competition with a test compound by MRM-based MS analysis	*m*/*z* and MS intensity	*K*_i_, *K*_on_, *K*_off_	Low
	Native MS	Detect intact protein–ligand complexes in the gas phase by MS	*m*/*z* and MS intensity	*K*_d_, stoichiometry (*n*)	Low
Stability-based assay	DSF	Detect changes in protein fluorescence over a temperature gradient	Fluorescence intensity	*T*_m_	Medium
	DLS	Measure changes in the protein aggregate size based on static light scattering properties over a temperature gradient	Light scattering intensity	*T*_agg_	Medium
Cell signaling assay	GTPγS	Detect ^35^S-GTPγS binding to GPCR-expressing cell membranes as a result of receptor activation	Radioactivity	EC_50_, IC_50_	High
	cAMP	Detect cellular levels of cAMP coupled to Gα_s_ or Gα_i_ activation	Luminescence/fluorescence	EC_50_, IC_50_	High
	Ca^2+^	Detect cellular levels of free Ca^2+^ coupled to Gα_q/11_ or Gα_15/16_ activation	Fluorescence	EC_50_, IC_50_	High
	IP3/IP1	Detect cellular levels of IP3 coupled to Gα_q_ or Gα_i_ activation	Fluorescence	EC_50_, IC_50_	High
	Luciferase reporter gene	Measure reporter gene transcriptional activity downstream of receptor activation	Luminescence	EC_50_, IC_50_	High
	β-arrestin recruitment	Detect cellular levels of β-arrestin along with receptor endocytosis	Luminescence	EC_50_, IC_50_	Medium–high

*DEL* DNA-encoded library, *SPR* surface plasmon resonance, *MST* microscale thermophoresis, *ALIS* automated ligand identification system, *UF* ultrafiltration, *FAC* frontal affinity chromatography, *DSF* differential scanning fluorimetry, *DLS* dynamin light scattering

*ALIS* is currently the most prevailing MS-based technique employed in pharmaceutical companies for HTS of large-scale synthetic compound libraries.^256,261,270–273^ This system integrates size exclusion chromatography for isolating protein–ligand complexes and reverse-phase chromatography for dissociating bound ligands, which are then identified by high-resolution MS. Not surprisingly, the application of ALIS to ligand screening for GPCRs substantially lagged behind soluble proteins due to difficulties in obtaining membrane receptors of sufficient purity and stability. The earliest application was ligand screening for M2R,^274^ in which purified M2R was incubated with a 1500-compound pool in each round of affinity selection. After screening a total of 350,000 compounds, one orthosteric antagonist and one allosteric modulator were identified for AChR. Later on, a similar strategy was implemented to screen ligands for CXCR4 using two libraries comprised of 48,000 and 2.75 million compounds, respectively. Each reaction consumed 250 ng purified receptor incubated with a pool of 100 or 2000 compounds. Out of the 362 primary hits, 34 were subsequently confirmed to be new antagonists.^262^

**Fig. 11:**
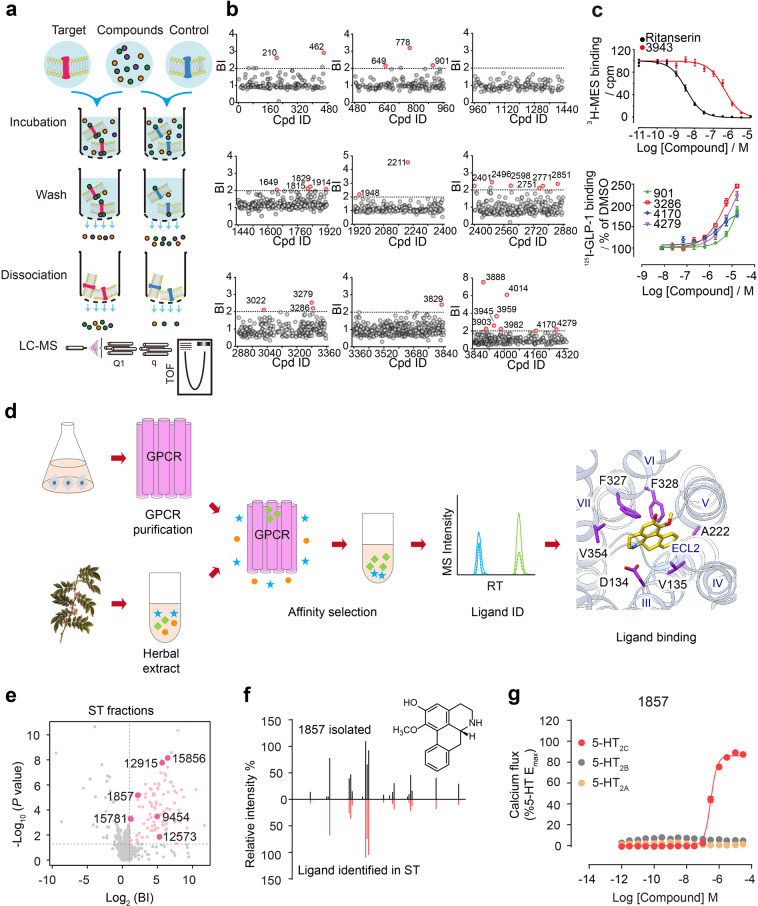
Developing affinity MS approaches for GPCR ligand screening. **a** Experimental workflow of membrane-based affinity MS. **b** Membrane-based affinity MS screening of 4333 compounds split into 9 cocktails against GLP-1R. Initial hits are indicated by red dots, while gray dots represent negatives. **c** Binding of one new ligand to 5-HT_2C_ (upper) and four new ligands to GLP-1R (lower) were validated by a radioligand-binding assay. **d** Experimental workflow of affinity MS-based screening of natural herb extracts. **e** Initial hits from screening fractionated herbal extracts toward 5-HT_2C_. Aporphines are annotated with larger pink dots. BI binding index. **f** Structural validation of 1857 by MSMS analysis. **g** 1857 displayed selective agonism at 5-HT_2C_. Source: adapted from Qin et al.^266^ and Zhang et al.^282^

*Membrane-based affinity MS* developed by Shui’s group enables ligand screening toward wild-type active GPCRs embedded in the cell membrane.^266^ It features isolation of membrane fractions from cells expressing a GPCR at high yield and incubation of the cell membrane with a compound cocktail, thus keeping the receptor in its native conformation and eliminating the need of protein purification. Compounds associated with the receptor were then released and subjected to high-resolution MS for structural assignment (Fig. 11a). Each incubation consumed about 2 µg membrane-embedded GPCR protein with a pool of 480 compounds. Primary hits were selected based on the binding index (BI) derived from quantitative MS signals used to distinguish putative ligands from non-specific binders (Fig. 11b). Screening a small compound library with this approach led to the discovery of an antagonist for the 5-HT_2C_ receptor and four PAMs for GLP-1R that are not reported previously^266^ (Fig. 11c).

More recently, the same team devised another affinity MS strategy that enabled screening of 20,000 compounds in one pool.^275^ Specifically, they modified the workflow by performing iterative rounds of affinity selection for compounds associated with A_2A_R. Similar to the previously described single-round affinity MS screening assay, quantitative measurement of BI renders detection of high-affinity ligands in this experiment. By comparing the selection of 16 benchmark A_2A_R ligands from screening compound pools of 480-mix, 2400-mix, 4800-mix, and 20K-mix, they demonstrated that this accelerated affinity MS screening approach, using either the purified receptor or receptor-expressing cell membranes, allowed detection of most high-affinity A_2A_R ligands (*K*_d_ or *K*_i_ <5 µM), and significant reduction of protein consumption and MS instrument time.^275^ Three new antagonists for A_2A_R were identified as a result. It is likely that the throughput of this method could be further increased to assay close to or above 1 million compounds in one pool.^275^

The affinity MS technique has been widely employed to fish out and identify putative ligands toward various enzyme targets from complex extracts of natural products, which could promote lead discovery from TCM.^276–281^ Indeed, this technique was successfully extended to GPCR ligand screening from herbal extracts. It involved the optimization of receptor construct and integration of affinity MS with metabolomics data mining workflow for sensitive and accurate ligand identification^282^ (Fig. 11d). After screening a panel of herbal extracts, a naturally occurring aporphine compound (1857) displaying strong subtype selectivity for 5-HT_2C_ without affecting 5-HT_2A_ or 5-HT_2B_ was discovered (Fig. 11e–g). Moreover, this new lead exhibited exclusive bias toward G protein signaling and showed in vivo efficacy for food intake suppression and weight loss.^282^

Although not directly applied to GPCRs, a previously reported cell-based assay vascular endothelial growth factor receptor 2 (VEGFR2) is interesting.^283^ It used a special one-bead-one-compound library of peptoids and cells overexpressing VEGFR2. Beads bound to the color-coded VEGFR2-expressing cells were selected under fluorescence microscopy and the attached ligands decoded by tandem MS analysis. Hits with low μM affinity to the soluble VEGFR2 ectodomain were identified subsequently. We envision that these membrane-based or cell-based screening platforms will make a major impact on GPCR drug discovery, especially when they are fully integrated.

*Competitive MS binding assay* employs a non-radioactive ligand to compete the binding of a test compound to a protein target. It resembles radioligand-binding assays but avoids the use of radioisotope.^284–286^ When assaying, the marker ligand liberated from the target is measured by an multiple reaction monitoring-based MS method of high sensitivity and selectivity for compound detection. Not only established for a number of transporters and ion channels,^287–289^ this approach is equally effective in addressing GPCRs as recently exemplified on A_1_AR/A_2A_R and DRD1/2/5.^267,285,290,291^ It was shown that unlabeled marker compounds could substitute their radiolabeled counterparts in all types of ligand-binding characterization studies, including saturation, displacement, dissociation, and competitive association, yielding results in excellent accordance with classic radioligand-binding assays.^267,290^

## Emerging opportunities and prospects

Recent scientific and technological advancements in GPCR biology have provided an enormous amount of information that will benefit our current and future efforts in rational drug design. Integration and refinement of massive data by artificial intelligence is a clear direction to guide both virtual and experimental screening of efficacious therapeutic agents with new scaffolds and of novel chemotypes for all classes of GPCRs.

However, as described in this review, factors that influence GPCR drug discovery include, but not limited to, therapeutic target, chemical diversity, mechanism of signaling, ligand-binding site, mode of action, clinical indication, polypharmacology, etc. Future opportunities may arise from: (i) de-orphanization of orphan GPCRs to provide novel targets; (ii) new indication for drug intervention via discovery and/or repurposing efforts; (iii) development of lead compounds targeting classes B2 and F GPCRs to address unmet medical needs; and (iv) validation of polypharmacology may lead to improved drug therapies.

## Supplementary information

Supplemental material

Table S1

Table S2
