# Supplementary material for: G protein-coupled receptors: structure- and function-based drug discovery
Source: Signal Transduct Target Ther. 2021 Jan 8;6:7. doi: 10.1038/s41392-020-00435-w (PMC7790836; doi:10.1038/s41392-020-00435-w)
Supplement: Supplementary file 1 — Supplemental material [file 41392_2020_435_MOESM1_ESM.docx]

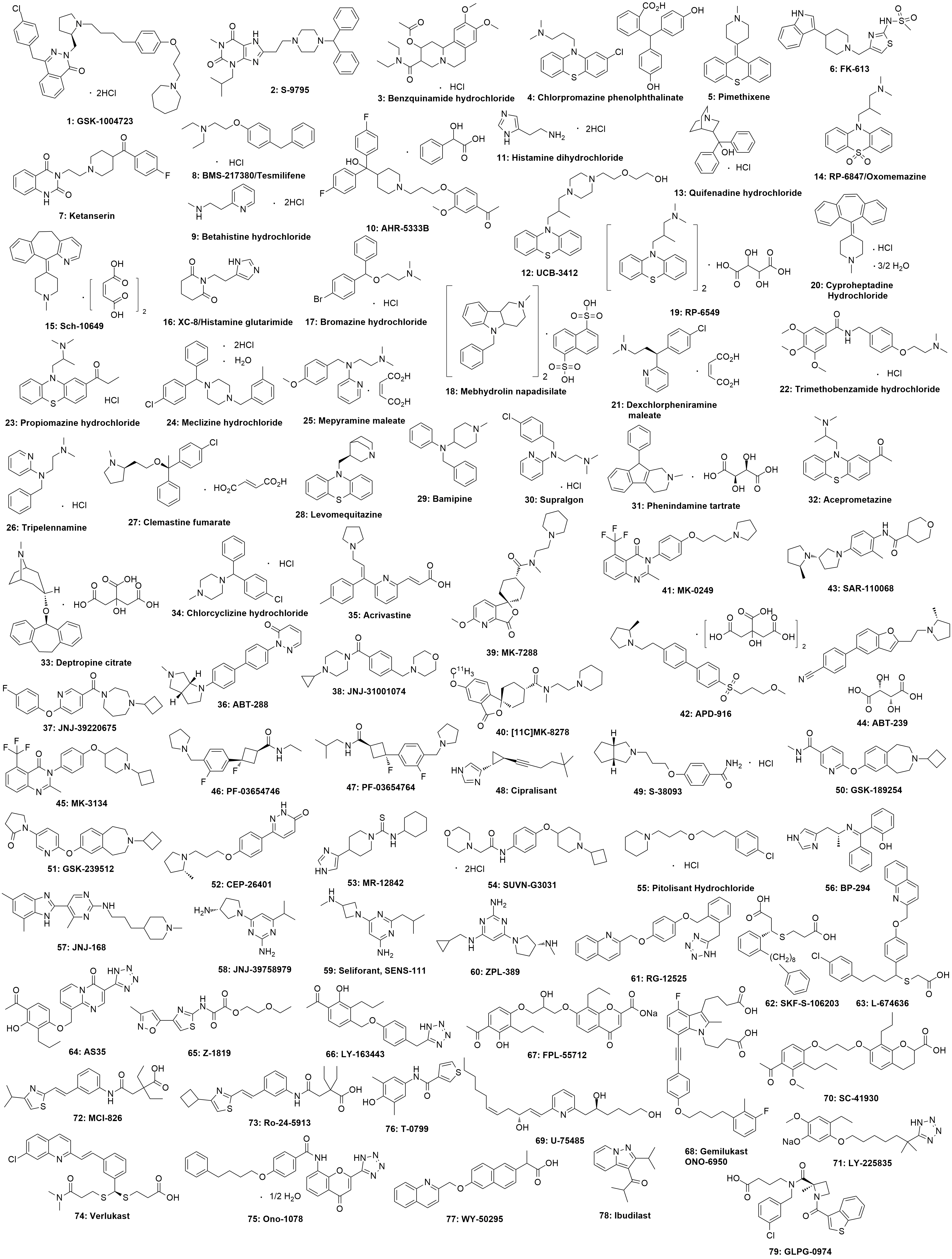


**Figure S1.** continued

**
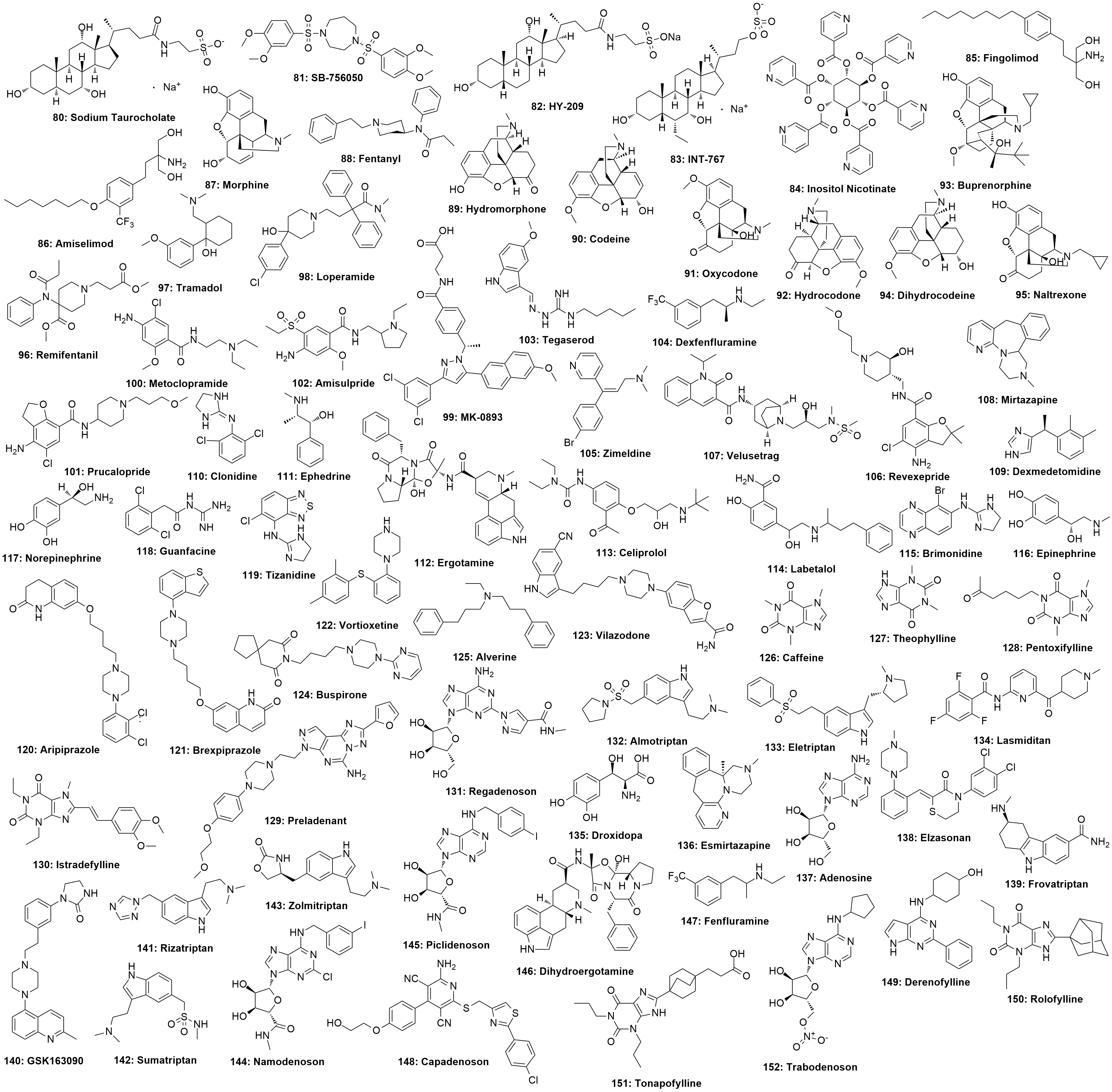
**

**Figure S1.** Structures of representative therapeutic agents targeting GPCRs approved or in clinical trials. See Table S3 for details.

**Table S1.** FDA approved drugs tageting GPCRs. See Table S1 excel file.

**Table S2.** Classification of GPCRs based on approved drugs on market. See Table S2 excel file.

**Table S3.** Major disease indications for GPCRs.

| **Agent (structure)** | **Target (mechanism)** | **Indication** | **Development status** |
| --- | --- | --- | --- |
| GSK-835726 | HRH1 and HRH3 (antagonist) | Seasonal allergic rhinitis | Phase II |
| GSK-1004723 (1) | HRH1 and HRH3 (antagonist) | Seasonal allergic rhinitis | Phase II |
| S-9795 (2) | HR and mAChR (antagonist), phosphodiesterase 1 (inhibitor) | Asthma | Phase II |
| Benzquinamide hydrochloride/emete-con^®^ (3) | HR and mAChR (antagonist) | Nausea and vomiting | Approved |
| Acetaminophen, clemastine fumarate, pseudoephedrine hydrochloride | HR (antagonist), COXs (inhibitor), α-adrenoceptors (agonist) | Headache, fever, allergic disease, cold | Approved |
| Chlorpromazine, phenolphthalinate (4) | HR, ADRA2 and DRD2 (antagonist) | Mania, schizophrenia, sedation, nausea, vomiting, depression, anxiety, anaesthesia, convulsion | Approved |
| Pimethixene (5) | 5-HT, mAChR and HR (antagonist) | Respiratory disturbance and cough | Approved |
| FK-613 (6) | 5-HT and HR (antagonist) | Urticaria, allergic rhinitis, asthma | Phase II |
| Ketanserin (7) | HRH1, ADR and 5-HT (antagonist) | Hypertension | Approved (hypertension);  Phase III (severe sepsis, septic shock) |
| BMS-217380, tesmilifene hydrochloride (8) | HR (antagonist) | Metastatic and recurrent breast cancer | Phase II |
| Betahistine/reboxetine (9) | HRH1 (agonist), HRH3 (antagonist), monoamine oxidase (substrate) | Weight gain caused by anti-psychotics, Meniere's syndrome | Phase II (obesity);  Phase IV (Meniere's syndrome);  Phase I (ADHD) |
| AHR-5333B (10) | HR (antagonist), mediator release (inhibitor) | Asthma | Phase I |
| JTE-350/histamine dihydrochloride (11) | NOX (inhibitor), HR (agonist) | AML | Approved (AML);  Phase I (middle ear disease, nasal allergy);  Phase II (seasonal allergic rhinitis);  Phase II (hypersensitivity) |
| Chlorpheniramine polistirex/hydrocodone polistirex | HR (antagonist), opioid receptor (agonist) | Respiratory disturbance, cough | Approved |
| WL-1017 | HR (antagonist) | Headache | Phase II |
| INT-0023 | HR (antagonist) | Allergic diseases | Phase I |
| UCB-3412/esucos^®^ (12) | HR (antagonist) | Sedation, nausea, vomiting, mental illness | Approved |
| WL-1011 | HR (antagonist) | Depression | Phase I |
| Quifenadine hydrochloride (13) | HR (antagonist) | Itching, urticaria, allergic rhinitis, dermatitis, angioedema | Approved |
| Oxomemazine (14) | HR (antagonist) | Itching, allergy, cough | Approved (itching, allergy);  Phase III (cough) |
| Sch-10649/optimine^®^ (15) | HR (antagonist) | Allergic rhinitis | Approved |
| XC-8/histamine glutarimide (16) | HR (modulator) | Seasonal allergic rhinitis, bronchial asthma, asthma | Phase II (seasonal allergic rhinitis);  Phase II (bronchial asthma);  Phase I (asthma) |
| Bromazine hydrochloride, ambrodyl^®^/ambrodil^®^ (17) | HR (antagonist) | Cough, allergic disease | Approved |
| Mebhydrolin napadisilate (18) | HR (antagonist) | Urticaria, itchy skin, allergic diseases | Approved |
| COL-118 | HR (antagonist) | Allergic rhinitis | Phase II |
| RP-6549/alimemazine tartrate (19) | HR (antagonist) | Urticaria, itchy skin | Approved |
| Cyproheptadine hydrochloride/periactin^®^ (20) | HR (antagonist) | Perennial allergic rhinitis, urticaria, conjunctivitis | Approved |
| Dexchlorpheniramine maleate/polaramine^®^/trimeton^®^ (21) | HR (antagonist) | Itchy, urticaria, allergic rhinitis, allergic conjunctivitis | Approved |
| Trimethobenzamide hydrochloride, tigan^®^ (22) | HR (antagonist) | Nausea, vomiting | Phase IV (Parkinson's disease, motor symptoms, akinesia, hypomobility, delayed levodopa onset);  Phase I (idiopathic Parkinson’s disease) |
| Propiomazine hydrochloride/largon^®^ (23) | HR (antagonist) | Insomnia | Approved |
| Meclizine hydrochloride/antivert^®^ (24) | HR (antagonist) | Nausea, vomiting, itchy skin, allergy, dizziness | Approved |
| Mepyramine maleate, anthisan^®^/pymafed^®^ (25) | HR (antagonist) | Itchy skin, cough, allergy, insomnia | Approved |
| Tripelennamine (26) | HR (antagonist) | Allergic disease | Approved |
| Clemastine fumarate/tavist^®^ (27) | HR (antagonist) | Urticaria, allergic rhinitis, itchy skin, eczema, allergic conjunctivitis | Approved |
| Betahistine hydrochloride/histalean^®^/serc^®^ (9) | HRH3 (antagonist) | Meniere's syndrome | Approved ( Meniere's syndrome);  Phase IV (gait or balance disorder problems);  Phase I (obesity, overweight, overnutrition);  Phase II (schizophrenia, schizoaffective disorder, schizophreniform disorder, bipolar I disorder, bipolar II, bipolar NOS, psychotic disorder not otherwise specified);  Phase II/completed (obesity);  Phase I (attention deficit disorder with hyperactivity) |
| Levomequitazine (28) | HR (antagonist) | Seasonal allergic rhinitis | Phase III |
| Bamipine (29) | HRH1 (antagonist) | Urticaria, skin itch, allergic disease | Approved |
| Supralgon^®^/suprastin^®^/synopen^®^ (30) | HRH1 (antagonist) | Bronchial asthma, allergic rhinitis, allergic conjunctivitis | Approved |
| Phenindamine tartrate (31) | HRH1 (antagonist) | Allergic disease | Approved |
| Aceprometazine (32) | HRH1 (antagonist) | Insomnia | Approved |
| Deptropine citrate (33) | HRH1 (antagonist) | Respiratory illness | Approved |
| Chlorcyclizine hydrochloride (34) | HRH1 (antagonist) | Nausea, vomiting, allergic disease | Approved |
| Acrivastine (35) | HRH1 (antagonist) | Seasonal allergic rhinitis, urticaria | Approved |
| ABT-834 | HRH3 (antagonist) | Cognitive disorder | Phase I |
| ABT-288 (36) | HRH3 (antagonist) | Cognitive deficits in schizophrenia, Alzheimer's disease, schizophrenia | Phase II (cognitive deficits in schizophrenia);  Phase II (Alzheimer's disease);  Phase II (schizophrenia) |
| JNJ-17216498 | HRH3 (antagonist) | Narcolepsy | Phase II |
| JNJ-39220675 (37) | HRH3 (antagonist) | Allergic rhinitis | Phase II |
| JNJ-31001074/bavisant (38) | HRH3 (antagonist) | Narcolepsy, ADHD | Phase II (excessive daytime sleepiness, Parkinson’s disease);  Phase II (ADHD) |
| MK-7288 (39) | HRH3 (inverse agonist) | Apnea, sleep | Phase I |
| [^11^C]MK-8278 (40) | HRH3 (inverse agonist) | Diagnostic reagent | Phase I |
| MK-0249 (41) | HRH3 (inverse agonist) | Alzheimer's disease, dementia, Paranoid schizophrenia, ADHD | Phase II (paranoid schizophrenia);  Phase II (ADHD);  Phase II (Alzheimer's disease) |
| SAR 152954 | HRH3 (antagonist) | Somnipathy | Phase I |
| SCH-497079 | HRH3 (antagonist) | Obesity, overweight, T2DM | Phase II |
| SAR-110894 | HRH3 (antagonist) | Alzheimer’s disease | Phase II |
| UCB-2892 | HRH3 (antagonist) | CNS disorder | Phase I |
| AZD-5213 | HRH3 (antagonist) | Diabetic neuropathy, Tourette syndrome, mild cognitive impairment, mild Alzheimer's disease | Phase II (painful diabetic neuropathy, diabetic neuropathies);  Phase II (Tourette syndrome);  Phase II (mild cognitive impairment, mild Alzheimer's disease) |
| APD-916 (42) | HRH3 (inverse agonist) | Narcolepsy with or without cataplexy | Phase I |
| SAR-110068 (43) | HRH3 (antagonist) | Sleep disorders, hypnosia | Preclinical |
| ABT-239 (44) | HRH3 (antagonist) | Cognitive disorder | Phase I |
| MK-3134 (45) | HRH3 (agonist) | Dementia | Phase I |
| SLS-010 | HRH3 (antagonist) | Hypnosia | Preclinical |
| PF-03654746 (46) | HRH3 (antagonist) | ADHD, narcolepsy, allergic rhinitis, schizophrenia | Phase II (ADHD);  Phase II (narcolepsy);  Phase II (allergic rhinitis);  Phase I (schizophrenia) |
| PF-03654764 (47) | HRH3 (antagonist) | Allergic rhinitis | Phase II |
| Cipralisant/GT-2331 (48) | HRH3 (antagonist) | ADHD, sleep disorders, cognitive disorders | Phase II |
| S-38093 (49) | HRH3 (antagonist) | Alzheimer's disease | Phase II |
| SCH-1681027 | HRH3 (antagonist) | Obesity, type 2 diabetes, allergic rhinitis | Pre-clinical |
| GSK-189254 (50) | HRH3 (antagonist) | Hyperalgesia | Phase I |
| GSK-239512 (51) | HRH3 (antagonist) | Schizophrenia, multiple sclerosis, Alzheimer's disease, mild cognitive impairment, dementia | Phase II (schizophrenia);  Phase I (multiple sclerosis);  Phase II (relapsing-remitting multiple sclerosis),  Phase II (Alzheimer's disease);  Phase I (mild cognitive impairment, dementia) |
| Irdabisant/CEP-26401 (52) | HRH3 (antagonist and inverse agonist) | Cognitive impairment | Phase I |
| MR-12842/ thioperamide (53) | HRH3 (antagonist) | Cognitive impairment, mental illness | Phase II |
| SUVN-G3031 (54) | HRH3 (antagonist) | Cognitive disorders, Parkinson's disease | Phase I (cognitive disorders) |
| Pitolisant hydrochloride (55) | HRH3 (inverse agonist) | Narcolepsy, Parkinson's disease | Approved (narcolepsy);  Phase II (schizophrenia);  Phase III (narcolepsy with cataplexy, excessive daytime sleepiness);  Phase III (obstructive sleep apnea);  Phase III (Parkinson's disease);  Phase I (renal impairment) |
| BP-294 (56) | HRH3 (agonist) | Peptic ulcer, asthma | Phase I |
| JNJ-168/toreforant (57) | HRH4 (antagonist) | Asthma, psoriasis | Phase II (psoriasis);  Phase I (hepatic impairment);  Phase II (asthma);  Phase I (rheumatoid arthritis) |
| JNJ-39758979 (58) | HRH4 (antagonist) | Rheumatoid arthritis, atopic dermatitis, asthma | Phase II (asthma);  Phase I (histamine induced itch);  Phase I (rheumatoid arthritis) |
| Seliforant/UR-63325/SENS-111 (59) | HRH4 (antagonist) | Tinnitus, dizziness, seasonal allergic rhinitis | Phase II |
| ZPL-389/PF-03893787 (60) | HRH4 (antagonist) | Atopic dermatitis | Phase II (atopic dermatitis);  Phase I (asthma) |
| RG-12525 (61) | LTRs (antagonist) | Asthma | Phase II |
| SKF-S-106203 (62) | LTRs (antagonist) | Asthma | Phase II |
| L-674636(63) | LTRs (antagonist) | Respiratory disease | Preclinical |
| CS-615 | LTRs (antagonist) | Asthma | Phase II |
| AS-35 (64) | LTRs (antagonist) | Asthma | Phase II |
| Z-1819 (65) | LTRs (antagonist) | Allergic diseases, asthma | Phase I |
| LY-163443 (66) | LTRs (antagonist) | Asthma | Phase I |
| FPL-55712 (67) | LTRs (antagonist) | Asthma | Phase II |
| Gemilukast/ONO-6950 (68) | CYSLTR2 (antagonist) | Asthma | Phase II |
| U-75485 (69) | LTB4Rs (antagonist) | Respiratory disease | Preclinical |
| SC-41930 (70) | LTB4Rs (antagonist) | Rheumatic diseases, psoriasis, inflammatory bowel disease | Phase II |
| LY-225835 (71) | LTB4Rs (antagonist) | Inflammation | Preclinical |
| MCI-826(72) | LTRs (antagonist) | Allergic diseases, asthma | Phase I |
| WY-46016 | LTRs (antagonist) | Allergic diseases, asthma | Unknown |
| Ro-24-5913/cinalukast (73) | LTRs (antagonist) | Allergic diseases, asthma | Phase II |
| MK-679/verlukast (74) | LTRs (antagonist) | Asthma | Phase II |
| Ono-1078/pranlukast hydrate (75) | LTD4Rs, LTC4Rs, LTE4Rs (antagonist) | Chronic sinusitis, perennial allergic rhinitis | Phase III (chronic sinusitis);  Phase III (perennial allergic rhinitis) |
| T-0799 (76) | LTRs (antagonist) | Inflammation | Preclinical |
| WY-50295 (77) | LTRs (antagonist) | Psoriasis, inflammation, asthma, arthritis | Phase II |
| SC-106 | LTRs (antagonist), prostaglandin synthase (inhibitor) | Rheumatoid arthritis | Phase II |
| Ibudilast (78) | Platelet-activating factor, LTRs (antagonist), cGMP-PDE (inhibitor) | Multiple sclerosis, chronic neuralgia, asthma | Approved (multiple sclerosis, chronic neuralgia, asthma);  Phase II (methamphetamine dependence, HIV infection);  Phase II (amyotrophic lateral sclerosis);  Phase II (opioid abuse, opioid dependence);  Phase II (opioid-related disorders);  Phase I (alcohol use disorder);  Phase I (migraine headache);  Phase II (medication overuse headache);  Phase II (diabetic neuropathy) |
| DS-3801 | MTLR (agonist) | Chronic stubborn constipation | Phase I |
| JTT-851 | FFAR1 (agonist) | T2DM | Phase II |
| GLPG-0974 (79) | FFAR2 (antagonist) | Ulcerative colitis | Phase II |
| ARI-3037MO | HCAR2, HCAR3 (agonist) | Hyperlipidemia, nonalcoholic steatohepatitis, hypertriglyceridemia | Phase II (hyperlipidemia);  Phase II (nonalcoholic steatohepatitis);  Phase II (hypertriglyceridemia) |
| Sodium taurocholate (80) | GPBAR1 (agonist) | Obesity, T2DM | Phase II |
| XL-475 | GPBAR1 (agonist) | T2DM, metabolic disorders | Preclinical |
| SB-756050 (81) | GPBAR1 (agonist) | T2DM | Phase II |
| HY-209/sodium taurodeoxycholic acid (82) | GPBAR1 (agonist) | Atopic dermatitis | Phase I |
| INT-767 (83) | GPBAR1 (agonist) | Hepatic fibrosis | Phase I |
| Inositol nicotinate (84) | HCAR2, HCAR3 (agonist) | Peripheral vascular disease, hyperlipidemia | Phase II (trichotillomania, hair pulling);  Phase IV (pediatric bipolar spectrum disorders) |
| Fingolimod (85) | S1PR2 (agonist) | Multiple sclerosis, relapsing-remitting multiple sclerosis, immune system disease, chronic inflammatory demyelinating polyradiculoneuropathy, primary progressive multiple sclerosis, schizophrenia, asthma, amyotrophic lateral sclerosis, stroke, renal insufficiency, Rett syndrome, chronic progressive multiple sclerosis, anaplastic astrocytoma, glioblastoma multiforme | Phase IV (multiple sclerosis);  Phase IV (relapsing-remitting multiple sclerosis);  Phase IV (immune system disease);  Phase III (chronic inflammatory demyelinating polyradiculoneuropathy);  Phase III (primary progressive multiple sclerosis);  Phase II (schizophrenia);  Phase II (asthma);  Phase II (amyotrophic lateral sclerosis);  Phase II (stroke);  Phase I (renal insufficiency);  Phase I (Rett syndrome);  Phase I (chronic progressive multiple sclerosis) |
| Amiselimod (86) | S1PR2 (modulator) | Relapsing-remitting multiple sclerosis, psoriasis, Crohn's disease, systemic lupus erythematosus | Phase II (relapsing-remitting multiple sclerosis);  Phase II (psoriasis);  Phase II (Crohn's disease);  Phase I (systemic lupus erythematosus) |
| Morphine (87) | OPRM1 (agonist) | Pain, cancer | Phase IV (pain);  Phase IV (cancer) |
| Fentanyl (88) | OPRM1 (agonist) | Osteoarthritis, knee | Phase IV |
| Hydromorphone (89) | OPRM1 (agonist) | Cancer | Phase IV |
| Codeine (90) | OPRM1 (agonist) | Pain | Phase IV |
| Oxycodone (91) | OPRM1 (agonist) | Back pain | Phase IV |
| Hydrocodone (92) | OPRM1 (agonist) | Pain | Phase IV |
| Buprenorphine (93) | OPRM1 (agonist) | Pain | Phase IV |
| Dihydrocodeine (94) | OPRM1 (agonist) | Pain | Phase IV |
| Naltrexone (95) | OPRM1 (antagonist) | Alcohol dependence | Phase IV |
| Remifentanil (96) | OPRM1 (agonist) | Kidney stone | Phase IV |
| Tramadol (97) | OPRM1 (agonist) | Inguinal hernia | Phase IV |
| Loperamide (98) | OPRM1 (agonist) | Diarrhea | Phase IV |
| Liraglutide | GLP-1R (agonist) | T2DM | Phase IV |
| Exenatide | GLP-1R (agonist) | T2DM | Phase IV |
| Lixisenatide | GLP-1R (agonist) | T2DM | Phase IV |
| Glucagon | GCGR (agonist) | Type 1 diabetes, hypoglycemia, diabetes mellitus | Phase IV (type I diabetes);  Phase III (hypoglycemia);  Phase III (diabetes mellitus) |
| PF-06291874 | GCGR (antagonist) | T2DM | Phase II |
| MK-0893 (99) | GCGR (antagonist) | T2DM | Phase II |
| SAR425899 | GCGR (agonist) | T2DM | Phase II |
| Teriparatide | PTH1R (agonist) | Osteoporosis, postmenopausal osteoporosis | Phase IV (osteoporosis);  Phase IV (postmenopausal osteoporosis) |
| Parathyroid hormone | PTH1R (agonist) | Osteoporosis | Phase IV |
| Abaloparatide | PTH1R (agonist) | Osteoporosis | Phase II |
| Metoclopramide (100) | HTR4 (agonist) | Nausea, angina pectoris, migraine disorder, post-operative nausea and vomiting, migraine with/without aura, premature birth, pregnancy, opioid dependence, gastroenteritis, prostate cancer, vertigo, vomiting, headache, acute myocardial infarction, neoplasm, gastroparesis, injury, pain | Phase IV (nausea);  Phase IV (angina pectoris);  Phase IV (migraine disorder);  Phase IV (post-operative nausea and vomiting);  Phase IV (migraine with/without aura);  Phase IV (premature birth);  Phase IV (pregnancy);  Phase IV (opioid dependence);  Phase IV (gastroenteritis);  Phase IV (prostate cancer);  Phase IV (vertigo);  Phase IV (vomiting);  Phase IV (headache);  Phase IV (acute myocardial infarction);  Phase III (neoplasm);  Phase III (gastroparesis);  Phase II (injury);  Phase II (pain) |
| Prucalopride (101) | HTR4 (agonist) | Constipation, chronic constipation, gastroparesis, ileus, dysphagia, liver disease | Phase IV (constipation);  Phase IV (chronic constipation);  Phase IV (gastroparesis);  Phase IV (ileus);  Phase II (dysphagia);  Phase I (liver disease) |
| Amisulpride (102) | HTR4 (agonist); HTR1A, HTR1B, HTR1D, HTR1E, HTR1F, HTR5A (antagonist) | Schizophrenia, schizoaffective disorder, movement disorder, post operative nausea and vomiting, chemotherapy-induced nausea and vomiting, bipolar disorder, major depressive disorder, obsessive-compulsive disorder | Phase IV (schizophrenia);  Phase IV (schizoaffective disorder);  Phase IV (movement disorder);  Phase III (post operative nausea and vomiting);  Phase II (chemotherapy-induced nausea and vomiting);  Phase II (bipolar disorder);  Phase I (major depressive disorder);  Phase I (obsessive-compulsive disorder) |
| Tegaserod (103) | HTR4 (agonist) | Constipation, chronic constipation, irritable bowel syndrome, dyspepsia, gastroesophageal reflux disease | Phase IV (constipation);  Phase IV (chronic constipation);  Phase IV (irritable bowel syndrome);  Phase III (dyspepsia);  Phase III (gastroesophageal reflux disease) |
| Dexfenfluramine (104) | HTR4 (agonist) | Obesity | Phase IV |
| Zimeldine (105) | HTR4 (antagonist) | Unipolar depression | Phase IV |
| Revexepride (106) | HTR4 (agonist) | Gastroesophageal reflux disease | Phase II (gastroesophageal reflux disease) |
| Velusetrag (107) | HTR4 (agonist) | Gastroparesis, chronic constipation, Alzheimer's disease | Phase II (gastroparesis);  Phase II (chronic constipation);  Phase I (Alzheimer's disease) |
| Mirtazapine (108) | ADRA2A, ADRA2C (antagonist) | Unipolar depression, major depressive disorder | Phase IV (unipolar depression);  Phase IV (major depressive disorder) |
| Dexmedetomidine (109) | ADRA2A, ADRA2B, ADRA2C (agonist) | Pain, tinnitus, hemorrhage, kidney disease, cancer, brain neoplasm, agitation, opioid dependence, ischemia reperfusion injury, post-operative sign or symptom, hypertension, gastric cancer, bradycardia, delirium, endometrium adenocarcinoma, Parkinson's disease, cholecystitis, obstructive sleep apnea, septic shock, interstitial cystitis, thoracic aortic aneurysm, hemorrhage, tinnitus, mandibular prognathia | Phase IV (pain);  Phase IV (tinnitus);  Phase IV (hemorrhage);  Phase IV (kidney disease);  Phase IV (brain neoplasm);  Phase IV (agitation);  Phase IV (opioid dependence);  Phase IV (ischemia reperfusion injury);  Phase IV (post-operative sign or symptom);  Phase IV (hypertension);  Phase IV (gastric cancer);  Phase IV (bradycardia);  Phase IV (delirium);  Phase IV (endometrium adenocarcinoma);  Phase IV (Parkinson's disease);  Phase IV (cholecystitis);  Phase IV (obstructive sleep apnea);  Phase IV (septic shock);  Phase IV (interstitial cystitis);  Phase IV (thoracic aortic aneurysm);  Phase IV (mandibular prognathia) |
| Clonidine (110) | ADRA2A, ADRA2B, ADRA2C (agonist) | Hypertension, agitation, pain, preeclampsia, neonatal abstinence syndrome, Tourette syndrome, ADHD, heart failure, opioid dependence, hip dysplasia, arterial occlusive disease, pregnancy, delirium, upper extremity fracture, hyperemesis gravidarum, diabetic neuropathy | Phase IV (hypertension);  Phase IV (agitation);  Phase IV (pain);  Phase IV (preeclampsia);  Phase IV (neonatal abstinence syndrome);  Phase IV (Tourette syndrome);  Phase IV (ADHD);  Phase IV (heart failure);  Phase IV (opioid dependence);  Phase IV (hip dysplasia);  Phase IV (arterial occlusive disease); Phase IV (pregnancy);  Phase III (delirium);  Phase III (upper extremity fracture);  Phase III (hyperemesis gravidarum);  Phase II (diabetic neuropathy) |
| Ephedrine (111) | ADRA2A, ADRA2B, ADRA2C (agonist) | Hypotension, gallstones, pregnancy, brain neoplasm, bradycardia, heart failure, obesity, hemorrhage, blood coagulation disease | Phase IV (hypotension);  Phase IV (gallstones);  Phase IV (pregnancy);  Phase IV (brain neoplasm);  Phase IV (bradycardia);  Phase IV (heart failure);  Phase III (obesity);  Phase II (hemorrhage);  Phase II (blood coagulation disease) |
| Ergotamine (112) | HTR1D, ADRA2A, ADRA2B, ADRA2C (agonist) | Migraine disorder, orthostatic hypotension | Phase IV (migraine disorder);  Phase I (orthostatic hypotension) |
| Celiprolol (113) | ADRA2A, ADRA2B, ADRA2C (antagonist) | Chronic obstructive pulmonary disease, vascular type Ehlers-Danlos syndrome, | Phase IV (chronic obstructive pulmonary disease);  Phase IV (Ehlers-Danlos syndrome, vascular type) |
| Labetalol (114) | ADRA2A, [ADRA2B](https://www.targetvalidation.org/target/ENSG00000274286), ADRA2C (antagonist) | Preeclampsia, hypotension, nicotine dependence, intracerebral hemorrhage, sinusitis, cocaine dependence, stroke | Phase IV (preeclampsia);  Phase IV (hypotension);  Phase II (nicotine dependence);  Phase II (intracerebral hemorrhage);  Phase II (sinusitis);  Phase II (cocaine dependence);  Phase II (stroke) |
| Brimonidine (115) | ADRA2A, ADRA2B, ADRA2C (agonist) | Ocular hypertension, glaucoma, open-angle glaucoma, low tension glaucoma, rosacea, prostate cancer, hyperemia, erythema, allergic conjunctivitis, dry eye syndrome, rhegmatogenous retinal detachment, macular degeneration, diabetic retinopathy | Phase IV (ocular hypertension);  Phase IV (glaucoma);  Phase IV (open-angle glaucoma);  Phase IV (low tension glaucoma);  Phase IV (rosacea);  Phase IV (prostate cancer);  Phase III (hyperemia);  Phase III (erythema);  Phase II (allergic conjunctivitis);  Phase II (dry eye syndrome);  Phase II (rhegmatogenous retinal detachment);  Phase II (macular degeneration);  Phase II (diabetic retinopathy);  Phase I (retinitis pigmentosa) |
| Epinephrine (116) | ADRA2A, ADRA2B, ADRA2C (agonist) | Asthma, pain, osteoarthritis, cardiac arrhythmia, hip dysplasia, bronchiolitis | Phase IV (asthma);  Phase IV (pain);  Phase IV (osteoarthritis);  Phase IV (cardiac arrhythmia);  Phase IV (hip dysplasia);  Phase IV (bronchiolitis) |
| Norepinephrine (117) | ADRA2A, ADRA2C (agonist) | Septic shock, hypotension, shock | Phase IV (septic shock);  Phase IV (hypotension);  Phase IV (shock) |
| Guanfacine (118) | ADRA2A, ADRA2B, ADRA2C (agonist) | ADHD, Tourette syndrome, hypertension, nicotine dependence, stroke, cocaine dependence | Phase IV (ADHD);  Phase IV (Tourette syndrome);  Phase III (hypertension);  Phase II (nicotine dependence);  Phase II (stroke);  Phase II (cocaine dependence) |
| Tizanidine (119) | ADRA2A, ADRA2C (agonist) | Upper limb spasticity | Phase IV |
| Aripiprazole (120) | HTR1A (partial agonist) | Social anxiety disorder schizophrenia, bipolar disorder, alcohol dependence, eating disorder, hyperprolactinemia, conduct disorder, schizoaffective disorder, major depressive disorder, anxiety, autism spectrum disorder | Phase IV (social anxiety disorder);  Phase IV (schizophrenia);  Phase IV (bipolar disorder);  Phase IV (alcohol dependence);  Phase IV (eating disorder);  Phase IV (hyperprolactinemia);  Phase IV (conduct disorder);  Phase IV (schizoaffective disorder);  Phase IV (major depressive disorder);  Phase IV (anxiety);  Phase IV (autism spectrum disorder) |
| Brexpiprazole (121) | HTR1A (partial agonist) | Bipolar disorder | Phase IV |
| Vortioxetine (122) | HTR1A (agonist) | Major depressive disorder, panic disorder, mental deterioration | Phase IV (major depressive disorder);  Phase IV (panic disorder);  Phase IV (mental deterioration) |
| Vilazodone (123) | HTR1A (partial agonist) | Unipolar depression, major depressive disorder | Phase IV (unipolar depression);  Phase IV (major depressive disorder) |
| Buspirone (124) | HTR1A (partial agonist) | Cannabis dependence, heroin dependence, hypothermia, dyspepsia, unipolar depression | Phase IV (cannabis dependence);  Phase IV (heroin dependence);  Phase IV (hypothermia);  Phase IV (dyspepsia);  Phase IV (unipolar depression) |
| Alverine (125) | HTR1A (antagonist) | Irritable bowel syndrome | Phase IV |
| Caffeine (126) | ADORA1, ADORA2A, ADORA2B, ADORA3 (antagonist) | Ischemia reperfusion injury, atherosclerosis, migraine disorder, common cold, energy expenditure, atrial fibrillation | Phase IV (ischemia reperfusion injury);  Phase IV (atherosclerosis);  Phase IV (migraine disorder);  Phase IV (common cold);  Phase IV (energy expenditure);  Phase III (atrial fibrillation) |
| Theophylline (127) | ADORA1, ADORA2A, ADORA2B, ADORA3 (antagonist) | Acute kidney injury, headache, asthma, chronic obstructive pulmonary disease, bronchiectasis, kidney failure, chronic kidney disease, altitude sickness, pseudohypoparathyroidism type 1A, apnea | Phase IV (acute kidney injury);  Phase IV (headache);  Phase IV (asthma);  Phase IV (chronic obstructive pulmonary disease);  Phase IV (bronchiectasis);  Phase IV (kidney failure);  Phase IV (chronic kidney disease);  Phase II (altitude sickness);  Phase II (pseudohypoparathyroidism type 1A);  Phase II (apnea) |
| Pentoxifylline (128) | ADORA2A, ADORA2B (antagonist) | Diabetic nephropathy, T2DM, chronic kidney disease, alcoholic hepatitis, nephrotic syndrome, endometriosis | Phase IV (diabetic nephropathy);  Phase IV (T2DM);  Phase IV (chronic kidney disease);  Phase IV (alcoholic hepatitis);  Phase III (nephrotic syndrome);  Phase III (endometriosis) |
| Preladenant (129) | ADORA2A (antagonist) | Parkinson's disease | Phase III |
| Istradefylline (130) | ADORA2A (antagonist) | Parkinson's disease | Phase III |
| Regadenoson (131) | ADORA2A (agonist) | Coronary artery disease | Phase III |
| Almotriptan (132) | HTR1D, HTR1F, HTR1B (agonist) | Migraine disorder | Phase IV |
| Eletriptan (133) | HTR1B, HTR1D, HTR1F (agonist) | Migraine disorder | Phase IV |
| Lasmiditan (134) | HTR1F (agonist) | Migraine disorder, drug dependence | Phase III (migraine disorder);  Phase I (drug dependence) |
| Droxidopa (135) | ADRA2B (agonist) | Parkinson's disease, hypotension, ADHD, fibromyalgia, orthostatic hypotension | Phase IV (Parkinson's disease);  Phase II (hypotension);  Phase II (ADHD);  Phase II (fibromyalgia);  Phase I (orthostatic hypotension) |
| Ergoloid | ADRA2B (antagonist) and HTR1B (agonist) | Vascular dementia | Phase II |
| Esmirtazapine (136) | ADRA2B (antagonist) | Insomnia | Phase III |
| Adenosine (137) | ADORA1, ADORA2B, ADORA3 (agonist) | Supraventricular tachycardia, atrial fibrillation, cancer, acute myocardial infarction, myocardial infarction, ST elevation myocardial Infarction, atrial fibrillation, chronic lymphocytic leukemia, coronary artery disease, angina pectoris | Phase IV (supraventricular tachycardia);  Phase III (atrial fibrillation);  Phase III (cancer);  Phase III (acute myocardial infarction);  Phase II (myocardial infarction);  Phase II (ST elevation myocardial infarction);  Phase II (atrial fibrillation);  Phase I (chronic lymphocytic leukemia);  Phase I (coronary artery disease);  Phase I (angina pectoris) |
| Elzasonan (138) | HTR1B, HTR1D (antagonist) | Major depressive disorder | Phase II |
| Frovatriptan (139) | HTR1B, HTR1D (agonist) | Migraine disorder, pain | Phase IV (migraine disorder);  Phase II (pain) |
| GSK163090 (140) | HTR1B, HTR1D (antagonist) | Depressive disorder, major depressive disorder | Phase II (depressive disorder);  Phase I (major depressive disorder) |
| Rizatriptan (141) | HTR1B, HTR1D (agonist) | Migraine disorder | Phase IV |
| Sumatriptan (142) | HTR1B, HTR1D (agonist) | Migraine disorder, migraine with aura | Phase IV (migraine disorder);  Phase III (migraine with aura) |
| Zolmitriptan (143) | HTR1B, HTR1D (agonist) | Migraine disorder | Phase IV |
| Namodenoson (144) | ADORA3 (agonist) | Non-alcoholic fatty liver disease, chronic hepatitis C virus infection, hepatocellular carcinoma | Phase II (non-alcoholic fatty liver disease);  Phase I (chronic hepatitis C virus infection);  Phase I (hepatocellular carcinoma) |
| Piclidenoson (145) | ADORA3 (agonist) | Keratoconjunctivitis sicca, glaucoma, rheumatoid arthritis, psoriasis, ocular hypertension | Phase III (keratoconjunctivitis sicca);  Phase II (glaucoma);  Phase II (rheumatoid arthritis);  Phase II (psoriasis);  Phase II (ocular hypertension) |
| Secretin | SCTR (agonist) | Autism, schizophrenia, obesity, chronic pancreatitis, dyspepsia | Phase III (autism);  Phase II (schizophrenia);  Phase II (obesity);  Phase I (chronic pancreatitis);  Phase I (dyspepsia) |
| Dihydroergotamine (146) | HTR1D (agonist) | Migraine disorder, migraine with/without aura | Phase IV (migraine disorder);  Phase I (migraine with/without aura) |
| Fenfluramine (147) | HTR1D (agonist) | Dravet syndrome | Phase III |
| BAY1067197 | ADORA1 (agonist) | Heart failure | Phase II |
| Capadenoson (148) | ADORA1 (partial agonist) | Atrial fibrillation | Phase II |
| Derenofylline (149) | ADORA1 (antagonist) | Congestive heart failure | Phase II |
| GW493838 | ADORA1 (agonist) | Neuropathic pain | Phase II |
| PBF-680 | ADORA1 (antagonist) | Asthma | Phase II |
| Rolofylline (150) | ADORA1 (antagonist) | Congestive heart failure, heart failure | Phase III ([congestive heart failure](https://www.targetvalidation.org/disease/EFO_0000373));  Phase II ([heart failure](https://www.targetvalidation.org/disease/EFO_0000373)) |
| Selodenoson | ADORA1 (agonist) | Atrial fibrillation | Phase II |
| Tecadenoson | ADORA1 (agonist) | Atrial fibrillation | Phase II |
| Tonapofylline (151) | ADORA1 (antagonist) | Congestive heart failure, renal insufficiency, liver disease | Phase II (congestive heart failure),  Phase II (renal insufficiency),  Phase I (liver disease) |
| Trabodenoson (152) | ADORA1 (agonist) | Open-angle glaucoma, ocular hypertension | Phase III (open-angle glaucoma),  Phase III (ocular hypertension) |
| Elsiglutide | GLP-2R (agonist) | Diarrhea | Phase II |
| Teduglutide | GLP-2R (agonist) | Short bowel syndrome, Crohn's disease, hyperlipidemia, liver disease, kidney disease | Phase IV (short bowel syndrome);  Phase II (Crohn's disease);  Phase II (hyperlipidemia);  Phase I (liver disease);  Phase I (kidney disease) |

Receptor abbreviations are according to IUPHAR. The disease indications for GPCRs listed above were identified manually from Open Targets Platform and cross-referenced with Pharmacodia and ChemBL databases. 5-HT, 5-hydroxytryptamine receptor (serotonin receptor); ADHD, attention-deficit, hyperactivity disorder; ADR, adrenergic receptors; ADRA2, alpha-2 adrenergic receptor subfamily with three members (ADRA2A, ADRA2B and ADRA2C); ADRA2A, adrenoceptor alpha-2A; ADRA2B, adrenoceptor alpha-2B; ADRA2C, adrenoceptor alpha-2C; ADORA1, adenosine A1 receptor; ADORA2A, adenosine A2a receptor; ADORA2B, adenosine A2b receptor; ADORA3, adenosine A3 receptor; AML, acute myelogenous leukemia; CYSLTR1, cysteinyl leukotriene receptor 1; CYSLTR2, cysteinyl leukotriene receptor 2; FFAR1, free fatty acid receptor 1; FFAR2, free fatty acid receptor 2; GCGR, glucagon receptor; GLP-1R, glucagon-like peptide-1 receptor; GLP-2R, glucagon-like peptide-2 receptor; GPBAR1, G protein-coupled bile acid receptor 1; HCAR2, hydroxycarboxylic acid receptor 2; HCAR3, hydroxycarboxylic acid receptor 3; HR, histamine receptor; HRH1, histamine receptor H1; HRH3, histamine receptor H3; HRH4, histamine receptor H4; HTR4, 5-hydroxytryptamine receptor 4; HTR1A, 5-hydroxytryptamine receptor 1A; HTR1B, 5-hydroxytryptamine receptor 1B; HTR1D, 5-hydroxytryptamine receptor 1D; HTR1E, 5-hydroxytryptamine receptor 1E; HTR1F, 5-hydroxytryptamine receptor 1F; HTR5A, 5-hydroxytryptamine receptor 5A; LTB4R, leukotriene B4 receptor; LTC4R, leukotriene C4 receptor; LTD4R, leukotriene D4 receptor; LTE4R, leukotriene E4 receptor; LTRs, leukotriene receptors; mAChR, muscarinic acetylcholine receptors (CHRM1-CHRM5, or M1R-M5R); MTLR, motilin receptor; NOX, NADPH oxidase; OPRM1, opioid receptor mu 1; PTH1R, parathyroid hormone receptor type 1; SCTR, secretin receptor; S1PR2, sphingosine-1-phosphate receptor 2; T2DM, type 2 diabetes mellitus.
